# Deep Impact of Random Amplification and Library Construction Methods on Viral Metagenomics Results

**DOI:** 10.3390/v13020253

**Published:** 2021-02-07

**Authors:** Béatrice Regnault, Thomas Bigot, Laurence Ma, Philippe Pérot, Sarah Temmam, Marc Eloit

**Affiliations:** 1 Pathogen Discovery Laboratory, Institut Pasteur, 75015 Paris, France; beatrice.regnault@pasteur.fr (B.R.); philippe.perot@pasteur.fr (P.P.); sarah.temmam@pasteur.fr (S.T.); 2 Bioinformatics and Biostatistics Hub, Computational Biology Department, Institut Pasteur, 75015 Paris, France; thomas.bigot@pasteur.fr; 3 Biomics Platform, C2RT, Institut Pasteur, 75015 Paris, France; laurence.ma@pasteur.fr; 4 Ecole Nationale Vétérinaire d’Alfort, 94700 Maisons-Alfort, France

**Keywords:** viral metagenomics, random amplification, sensitivity

## Abstract

Clinical metagenomics is a broad-range agnostic detection method of pathogens, including novel microorganisms. A major limit is the low pathogen load compared to the high background of host nucleic acids. To overcome this issue, several solutions exist, such as applying a very high depth of sequencing, or performing a relative enrichment of viral genomes associated with capsids. At the end, the quantity of total nucleic acids is often below the concentrations recommended by the manufacturers of library kits, which necessitates to random amplify nucleic acids. Using a pool of 26 viruses representative of viral diversity, we observed a deep impact of the nature of sample (total nucleic acids versus RNA only), the reverse transcription, the random amplification and library construction method on virus recovery. We further optimized the two most promising methods and assessed their performance with fully characterized reference virus stocks. Good genome coverage and limit of detection lower than 100 or 1000 genome copies per mL of plasma, depending on the genome viral type, were obtained from a three million reads dataset. Our study reveals that optimized random amplification is a technique of choice when insufficient amounts of nucleic acid are available for direct libraries constructions.

## 1. Introduction

Direct identification of viral genomes from clinical specimens using next-generation sequencing (NGS) remains challenging [1] because of the scarcity of viral genomic material due to the small size of the virus genomes and their low abundance in a high background of host and other microbial nucleic acids. This can lead to a relatively low detection sensitivity of 10^6^ genome equivalents per mL [2,3,4,5]. The diversity of virus structures and nucleic acid types has impaired the development of a unique viral metagenomics workflow, and the subsequent comprehensive detection and identification of viruses present in a clinical sample. Most of the published viral amplification protocols have been optimized for the detection of either DNA or RNA viruses [6,7]. In recent years, a number of groups have focused on RNA viruses because they account for the majority of emerging viruses [8,9]. One of the well-known major challenges of untargeted (agnostic) viral metagenomics is the overwhelming amount of host nucleic acids and other microbial genomic materials present in primary clinical specimens, whose content depends greatly on the type of specimen. A typical human cell can contain up to 10^6^ times as much DNA as a small virus. A single cell can contain up to 60,000 RNA molecules (excluding tRNA) [10]. For RNA sequencing libraries, most of this host background typically corresponds to human rRNA, mitochondrial RNA sequences, and highly expressed RNAs encoding proteins. Depletion of these human host sequences boosts the proportion of non-human microbial reads and thus improve the analytic sensitivity for pathogen detection [11]. Furthermore, metagenomics has the best sensitivity in paucicellular environments, such as cerebrospinal fluid rather than other biological samples like blood, respiratory samples or stool specimens [12].

To enrich the proportion of viral targets relative to host nucleic acids, and therefore improve analytical sensitivity, a typical workflow comprises the digestion of free nucleic acids that are not protected by viral capsids, or the precipitation of virus particles before viral nucleic acids extraction [13,14]. As a result, the absolute amount of remaining nucleic acids is very low, often non-measurable, and out of the specifications of the library preparation kits. It is, therefore, necessary to randomly amplify this virus-enriched fraction of nucleic acids. Among the random amplification techniques used in viral detection, methods based on sequence-independent single-primer amplification (SISPA) [15,16,17,18,19], multiple displacement amplification (MDA) [4] and linker amplification shotgun libraries (LASL) are the most frequently used [20]. Alternative amplification techniques, such as degenerate oligonucleotide primer PCR (DOP-PCR) [21] or multipleannealing and looping-based amplification cycles (MALBAC) [22] are often applied for single-cell whole-genome amplification. Nevertheless, these amplification steps prior to sequencing often lead to biases in the representation of certain viruses or viral families [23,24].

In this study, we compared the performances of virus detection of six methods designed for nucleic acids random amplification and subsequent sequencing library preparation with a method without pre-amplification on a panel of 26 known human viruses. To mimic a complex biological matrix, we further spiked a human plasma pool with a dilution of this virus panel, in order to evaluate the limit of detection of these methods from clinical samples. We also compared the impact of the nature of the nucleic acid extracts (i.e., total nucleic acid fraction or the RNA fraction) in the detection of viral sequences. Following this comparative study, we determined the limit of detection (LOD) of the best methods by spiking in plasma the WHO reference virus stocks (WRVS, quantified using digital PCR (dPCR)) and evaluated vertical and horizontal coverage of the viral genomes resulting from these random amplification and high-throughput sequencing.

## 2. Materials and Methods

### 2.1. Virus Panels

A sample comprising a suspension in PBS of 25 human viruses with different genomic and structural characteristics was purchased from the National Institute for Biological Standards and Controls (NIBSC code: 11-242-001). A representative of a linear ssDNA virus, Parvovirus B19 (NIBSC code: 12/208), was diluted to get Ct = 24 and added to the mix to form the virus multiplex reference panel (VMRP). The complete list of the 26 viruses is presented in Table S1. VMRP was either used undiluted or spiked into a human plasma pool at a volume-ratio 1:10.

WHO reference virus stocks (WRVS, Table S2A) were purchased from ATCC. Viruses composing this mix were quantified by ATCC regarding infectious virus titer (TCID50/mL) and genome copy number using digital drop PCR (Table S2B). Each of the five WRVS virus stocks was diluted into PBS, then pooled together at an equimolar genome concentration, and spiked to get a final concentration of 10^4^, 10^3^ or 10^2^ viral gc/mL of plasma pool. The plasma pool used in the two studies was derived from five human plasma samples from healthy blood donors purchased from ICAReB (Investigation Clinique et Accès aux Ressources Biologiques, Institut Pasteur, Paris, France).

### 2.2. Experimental Design

**Figure 1:**
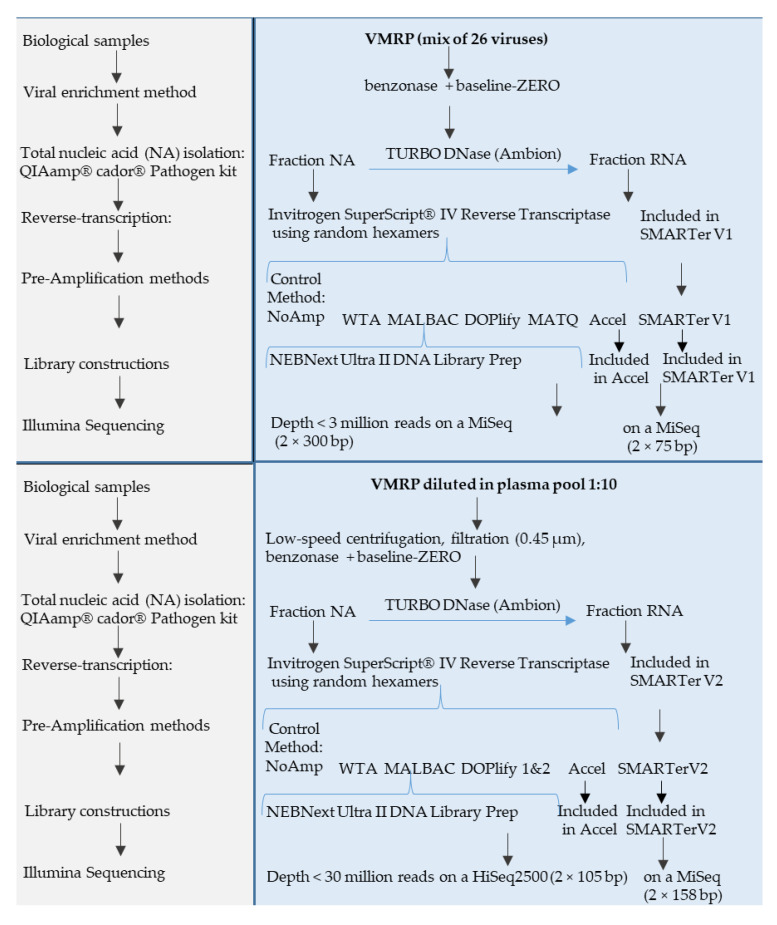
Experimental design with Virus Multiplex Reference Panel (VMRP). Six protocols including reverse transcription step, random pre-amplification step and subsequent library constructions were compared to a protocol without pre-amplification (NoAmp). A first experiment was carried out directly on a mix of 26 viruses representative of the diversity of viruses (VMRP) and the second experiment on VMRP diluted in a plasma matrix (ratio: 1/10). Both fractions, total nucleic acid fraction (NA) and RNA only, were used.

**Figure 2:**
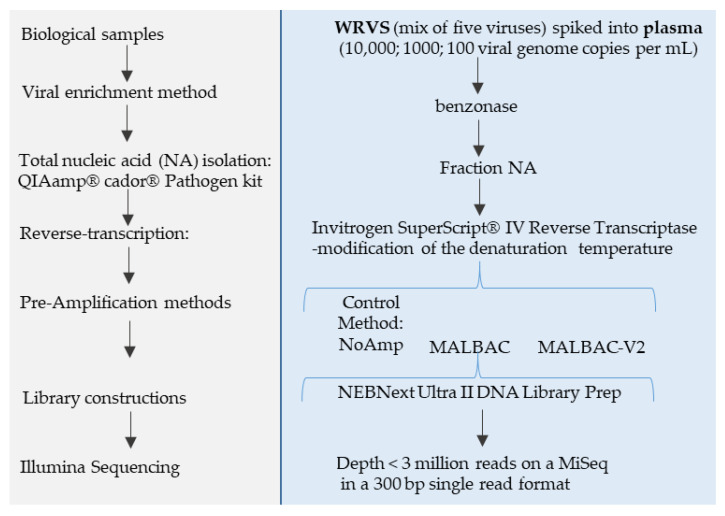
Experimental design with WHO reference virus stocks (WRVS). The experiment was designed to quantify the viral detection level of two MALBAC-based methods compared to the protocol without pre-amplification (NoAmp). The NA fraction of WRVS (mix of quantified reference virus stocks) was used at a final concentration of 10^4^, 10^3^ or 10^2^ viral gc/mL of plasma matrix.

A schematic overview of the experimental design for the evaluation of the different methods on the VMRP panel of 26 viruses, diluted or not in the plasma matrix, is given in Figure 1. Figure 2 shows the experimental design for the assessment of selected methods on the quantified WRVS reference virus stocks spiked into plasma. For both designs, each method was compared to the non-pre-amplified method (NoAmp). 

### 2.3. Nucleic Acid Extraction and Quantification

As summarized in Figure 1, VMRP was treated prior extraction with benzonase (Novagen, Madison, WI, USA) (5 U/µL) and baseline-ZERO (EUROMEDEX, Souffelweyersheim, France (1 U/µL) nucleases for 2 h at 37 °C, in order to digest unprotected nucleic acids. Enzymes were inactivated with a final concentration of 3 mM EDTA and heating for 10 min at 65 °C. A plasma sample was spiked with VMRP diluted at a ratio 1:10, then centrifuged at low speed, filtrated through Spin-X^®^ centrifuge tube filter 0.45 µM (cellulose acetate membrane) (Costar, WA, USA) then treated by nucleases as described above.

Other plasma samples derived from the same pool and spiked with WRVS at a final concentration of 10^4^, 10^3^ or 10^2^ viral gc/mLwere treated prior to extraction with benzonase (25 U/µL) for 2 h at 37 °C.

Total nucleic acids were extracted by the QIAamp^®^ Cador^®^ Pathogen kit (Qiagen, Courtaboeuf, France) with the substitution of carrier RNA by Linear Acrylamide (Ambion, Waltham, MA, USA) (10 µg per extraction). To get the RNA fraction, the extract was digested with TURBO DNase (Ambion) (10 U for 40 µL of nucleic acids) and purified with a Qiagen RNeasy Mini Kit.

All VMRP sample extracts were below the detection limit of the Qubit quantification system (LOQ = 200 pg for the dsDNA HS Assay Kit, Invitrogen, Waltham, MA, USA).

### 2.4. Amplification Methods

Before pre-amplification, reverse transcription (SuperScript IV Reverse Transcriptase, Invitrogen) using random hexamers was carried out on both total nucleic acids (NA fraction) and RNA fraction, except for SMARTer and MATQ methods, which included their own reverse transcription step. The methods of pre-amplification were compared to a method without amplification, referred to as the control method. Table 1 describes the principle of the different methods. Procedures can be found in Supplementary Materials.

### 2.5. Library Construction and Sequencing

Libraries were prepared following the instructions of the kit manufacturer for both SMARTer and Accel methods. For the other methods, the NEBNext Ultra II DNA Library Prep kit (New Englands Biolabs, Evry, France) for Illumina was chosen because it allows a broad range of input amounts, from 500 pg to 1 µg DNA. Our strategy was to construct the different libraries from the maximum amount of amplified nucleic acids available, with an upper limit of 1 µg DNA. Except for SMARTer libraries, DNA samples were fragmented by Covaris M220 Focused-Ultrasonicator (Covaris Ltd, Brighton, UK) using microTUBE-15. The adaptor concentration, the size selection post-ligation or clean-up only, and the PCR cycle number were adjusted according to the input material following the manufacturer recommendations. Libraries were analyzed for size distribution using the High Sensitivity DNA Kit (Agilent Technologies, Santa-Clara, CA, USA) on a Bioanalyser Instrument. The individually indexed libraries were quantified using Qubit HS DNA and pooled at equal molar quantity. Sequencing was carried out on Illumina MiSeq platform at a depth of three million reads for crude VMRP and for WRVS spiked in plasma, and on HiSeq2500 at a depth of 30 million reads for the VMRP diluted in the plasma matrix.

### 2.6. Data Analysis

Raw reads were processed with an in-house agnostic bioinformatics pipeline, as previously described [28], which includes quality check and trimming, read normalization (using BBNorm with k-mer target normalization depth = 100), de novo assembly, open reading frames (ORF) prediction (https://doi.org/10.6084/m9.figshare.7588592), and Diamond-blastp similarity search against the protein Reference Viral Database (RVDB-prot [29]) followed by the validation of viral taxonomic assignment by Diamond-blastp search against the whole protein NCBI/nr database (release 01 November 2019), and a final search against the whole NCBI/nt nucleotide database (release 15 August 2019) using blastn. The quantification of abundance of each viral taxon was obtained by summing the length (in nucleotides) of all sequences being associated to this taxon, weighted by the k-mer coverage of each contig. This metric, referred as WNCS for weighted number of contigs and singletons, is a global indicator of the viral genome fraction (horizontal coverage) and depth coverage (vertical coverage), adjusted according to the sequencing length of the reads.

Genome fraction is defined as the percentage of bases that align to the reference genome. Viral genome fractions were obtained by mapping against the reference genomes using CLC Genomics Workbench Version 9.5.3 Qiagen, with the following parameters: length fraction of 0.8 and similarity fraction of 0.9 for the whole study. Reference viral genomes used for read mapping of WRVS are presented in Table S3.

## 3. Results

### 3.1. Evaluation of Random Amplification Protocols Compared to a Control Method without Pre-Amplification for the Identification of 26 Viral Genomes (VMRP) in PBS

#### 3.1.1. DNA Yields After Random Amplification

The DNA yields obtained by random amplification of NA and RNA fractions from the same extracts of non-quantifiable nucleic acids are shown in Table 2.

The highest amounts of DNA product were achieved with WTA and MALBAC, both for NA and RNA fractions. The number of cycles of quasi-linear pre-amplification and amplification used for MALBAC were respectively 12 and 25, as the default number of cycles (8 and 17) did not allow to amplify the RNA fraction above the negative control (PBS). In presence of plasma, default number of cycles were used and generated a smaller amount of amplified product.

#### 3.1.2. VMRP Sequencing Metrics

Sequencing of the libraries of VMRP treated by the seven methods generated an average of 2,476,120 total reads (SD = 590,577) in NA fraction and an average 2,106,476 total reads (SD = 1,412,552) in RNA fraction after quality preprocessing (Table 2).

Replicated reads (redundancy) are usually introduced during library sequencing and are cumulated with those generated during the previous random amplification step, if any. For a given number of reads, the degree of replication has an impact on the viral genome fraction obtained. Read mapping of target viruses showed that the percentage of replicated reads was variable between methods. The highest percentage was observed for RNA viruses with SMARTerV1 method (respectively 33% and 54% for NA and RNA fractions). The Accel method showed an intermediate percentage of replicated reads (respectively 27% and 36% for NA, and RNA fractions). Both methods included their own library preparation that required respectively 16 and 17 cycles of final PCR. The other libraries, all constructed using the NEBNext kit, required less cycles of final PCR (three cycles for MALBAC, DOPlify and MATQ, and 11 cycles for NoAmp) and showed the lowest number of replicated reads. Among these libraries, DOPlify reached about 14% replicated reads, suggesting the direct impact of the pre-amplification step within the library construction.

#### 3.1.3. Viral Sequences Identified by In-House Agnostic Bioinformatics Pipeline

**Figure 3:**
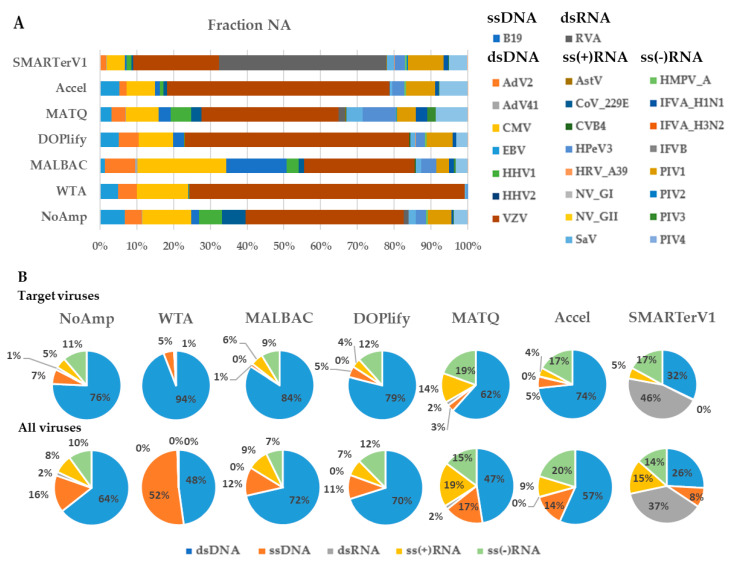

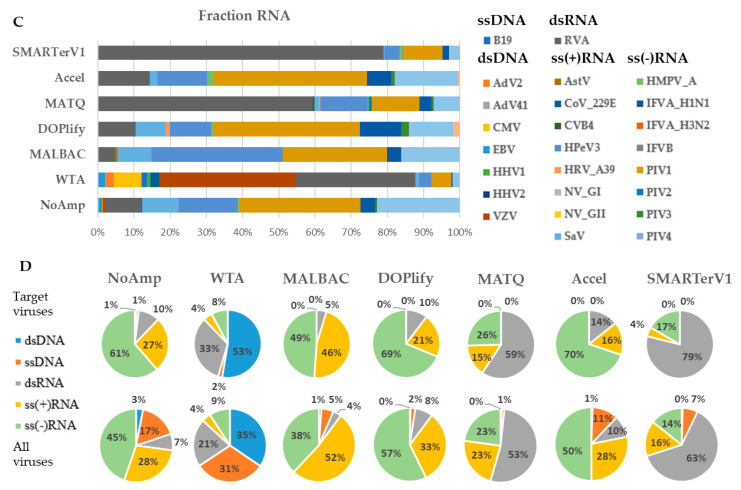
Proportion of spiked viruses (upper panels (**A**,**C**)) and viral genomic groups (lower panels (**B**,**D**)) in the VMRP based on the weighted contigs and singletons (WNCS). (**A**,**B**) In the NA fraction; (**C**,**D**) in the RNA fraction. Comparison of viral genomic groups is shown for target viruses (top) and for all viruses (bottom).

The weighted number of contigs and singletons parameter (WNCS) was used as readout to compare the proportion of the five genomic groups of viruses as defined by the Baltimore classification of viruses (dsDNA, ssDNA, dsRNA, ssRNA(+) and ssRNA (−)) in each fraction for all methods (Figure 3). Both target viruses and viruses arising from normal plasma virome or laboratory reagents were taken into account. Discriminating both sources of viruses was not the focus of this study, but this global picture allows showing some trends.

In the NA fraction (Figure 3B) for all viruses, except for SMARTerV1 that is dedicated to RNA, the dsDNA group was the most represented (47–72%). The ssDNA group represented 11–17% of the total counts, except for WTA that reached 52%. Accel, DOP, and MALBAC amplification methods failed to detect dsRNA viruses whereas SMARTerV1 showed the highest percentage of dsRNA viruses (37%). NoAmp and MATQ detected only 2% of dsRNA viruses among total viral sequences. All methods, except WTA, detected 7–19% and 7–20% sequences corresponding to the ssRNA(+) and the ssRNA(-) groups, respectively.

In the RNA fraction (Figure 3D), dsRNA viruses detected by SMARTerV1 and MATQ represented respectively 63% and 53% of total counts, unlike other methods (between 4% and 10%).

It was verified that the majority of the detected dsRNA viruses corresponded to the spike virus, the rest being Kadipiro virus, a known contaminant of nucleic acid extraction spin columns [30]. For the other methods, they represented 28–52% of the total for the ssRNA(+) group and 38–57% for the ssRNA(−). SMARTerV1, which only amplifies RNA, was able to detect transcripts of dsDNA viruses in NA fraction, originating from non-purified virus preparation. However, they were unexpectedly not found in the RNA fraction. We assume that this loss of sensitivity is due to the treatment of NA fraction with the TURBO DNase and column purification to obtain the RNA fraction.

Comparison with WNCS of spiked viruses shows that the proportion of non-spiked ssDNA viruses increased more specifically in fraction NA and especially with WTA. This viral group includes *Microviridae*, CRESS virus, *Circoviridae*, Parvovirus NIH-CQV, *Anelloviridae*, *Parvoviridae* (other than B19). The dsDNA viruses identified with all methods belongs to Caudovirales, *Mimiviridae*, and *Phycodnaviridae*. *Iridoviridae* were detected only with NoAmp, Accel and SMARTer. The ssRNA(+) virus, Bovine viral diarrhea virus 1 (Pestivirus A, *Flaviviridae*), a known contaminant of calf serum used in cell culture, was detected by all methods in both fractions, except for WTA.

The distribution of WNCS of targeted or untargeted viruses is shown as a boxplot (Figure S1). The best proportions of nucleotides assigned to viruses were obtained by MALBAC and MATQ for the NA fraction, both above the non-pre-amplified method. SMARTerV1 and Accel showed a similar profile, with a global lower efficiency than the non-pre-amplified method with reference to RNA viruses (SMARTer) or all viruses (Accel). WTA showed widely dispersed data.

For the RNA fraction the same trend was observed, with more dispersed data and the highest WNCS obtained for MATQ.

#### 3.1.4. Analysis of the Length of Contigs per Method

The length of the contigs is an important criterion for the evaluation of methods, as it has a direct impact on the capability to identify distant sequences and to optimize horizontal coverage. In this study, the contigs were all generated by de novo MEGAHIT assembling from the same nucleic material. The size distribution of contigs for all methods and both fractions is shown as a boxplot in Figure S2. In the NA fraction, the largest contigs were obtained for NoAmp, followed by MALBAC (for example, a contig of 32,759 bp of HHV-3 for NoAmp). In the RNA fraction, the largest contigs were obtained by NoAmp, followed by MATQ (for example, a contig of 6912 bp of PIV-4 was obtained for NoAmp).

#### 3.1.5. Viral Genome Fraction Analysis of VMRP

**Figure 4:**
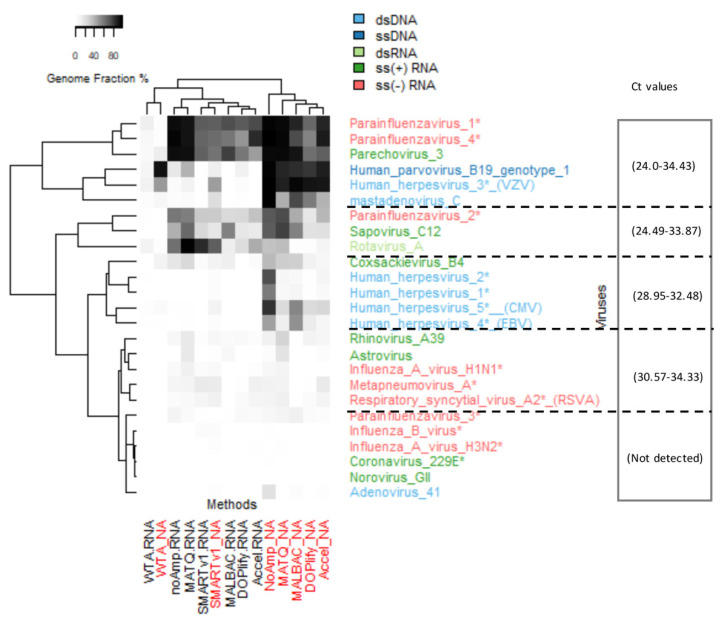
Heatmap of whole genome coverage of viruses of VMRP from seven methods in NA and RNA fractions. The genome fraction for each virus is in row and methods for both NA and RNA fractions are in column. Viruses are color-coded according to their genomic group (dsDNA, ssDNA, dsRNA, ss(+) RNA and ss(-) RNA). An asterisk * indicates the non-enveloped viruses. Norovirus GI detected by no method was removed. Ranges of Ct value previously determined [31] were reported per viral cluster.

The heatmap of genome fractions of targeted viruses presented in Figure 4 highlighted two clusters corresponding to NA and RNA fractions, except for the WTA and SMARTerV1 methods for which both fractions clustered together. In the case of SMARTer, this is explained by a comparable level of detection and coverage of the same RNA viruses in both fractions, while WTA had the lowest level of virus detection in both fractions. Within each fraction, Accel, DOPlify and MALBAC constituted a group of methods with close results. NoAmp and MATQ clustered together for the RNA fraction.

As expected, the NA fraction allowed a better genome coverage of DNA viruses (B19, Adv2, herpesviruses). The dsRNA virus was better covered in the RNA fraction.

The largest number of identified target viruses in the NA fraction was obtained by the pre-amplification-free control method (24 viruses) followed by SMARTer (despite its dedication to RNA viruses) and MATQ (Table 2). On the RNA fraction, 15 out of 18 target RNA viruses were identified by MATQ method, followed by the SMARTerV1 and the NoAmp methods, both with 11 viruses. The WTA method, despite it produced a high amount of amplified DNA, detected the lowest number of viruses. Table S4 reports the percentage of genome fractions and WNCS for detected viruses per method and fraction. Figure S3 shows the comparison of cumulative percentage of genome fractions for each DNA virus (A) and RNA virus (B) with the seven methods in both fractions. Most of RNA viruses were detected at comparable levels in both fractions.

There was no relationship regarding the ratio of mapped viral reads and the genome fraction covered by the reads. Indeed, MALBAC allowed the highest percentage of mapped target virus to total reads for the NA fraction (20.7%) (Table 2) while NoAmp detected only 1.35% viral reads in the same fraction. However, these methods detected respectively 17 (MALBAC) and 24 viruses (NoAmp).

#### 3.1.6. Recovery of Genomes of Diverse Virus Types and Comparison with Real-Time PCR

An estimate by qPCR of each viral genome abundance was previously described and six viruses among the VRMP were not detectable [31]. The threshold Cycle (Ct) (Table S1) are reported in the heatmap of genome fraction in Figure 4.

The most sensitive methods (NoAmp, MATQ, and SMARTerV1 in NA fraction) were able to detect all viruses detected by qPCRs. In addition, they also detected reads from some viruses not detected by qPCR: AdV41 (genome fraction 20% for NoAmpNA), PIV-3 (genome fraction 12%) and few reads of CoV 229E, IFVA H3N2, and IFVB. Thus, some NGS methods (especially NoAmp and SMARTer) show better sensitivity compared to qPCR, even using a relatively low sequencing depth (around 3 million). Some RNA viruses were detected by most protocols in both fractions (PIV-1 (Ct 34.43), PIV-4 (Ct 31.83), HPeV3 (Ct 29.35)).

Despite a high horizontal genome coverage, the methods revealed different coverage patterns. As an example of a virus detected by most methods in both fractions (WTA was omitted due to insufficient coverage), the genome coverages of PIV-1 were compared (Figure S4). PIV-1 is a negative single stranded RNA linear genome, 15.6 kb in size, encoding eight proteins and was previously detected at high Ct (Ct 34.43). The highest genome fraction was achieved in the NA fraction by NoAmp (98.2%) and MATQ (97.02%), then by MALBAC (87.35%) and Accel (87.13%) (Table S4). In the RNA fraction, NoAmp reached 95.5% and MATQ 93.2% of genome length coverage. The coverage along the genome of this virus was interrupted by numerous gaps with SMARTer and Accel methods.

Of note, all methods, including the NoAmp control, generated an over-coverage of the gene encoding the phosphoprotein in both fractions (Figure S5). To investigate whether this was due to an amplification bias or to the inherent nature of the virus (*Paramyxoviridae* viruses are characterized by a transcriptional gradient of mRNAs decreasing from 3′ to 5′ genes), the mapping of reads produced with the directional method SmarterV1 was analyzed to distinguish between genomic RNA and mRNA/antigenomic RNA. All reads R1 (representative of the genomic strand) were mapped as the same sense than the original RNA, and all reads R2 mapped as sequences antisense to the original RNA in both fractions, according to the sequencing protocol. This indicates that most reads corresponded to transcripts and that the over-coverage region observed in the phosphoprotein gene did not originate from an amplification bias, but was more likely due to the transcriptional pattern of this paramyxovirus.

### 3.2. Effect of a Complex Host Matrix on the Sensibility of the Different Methods of Random Amplification

**Figure 5:**
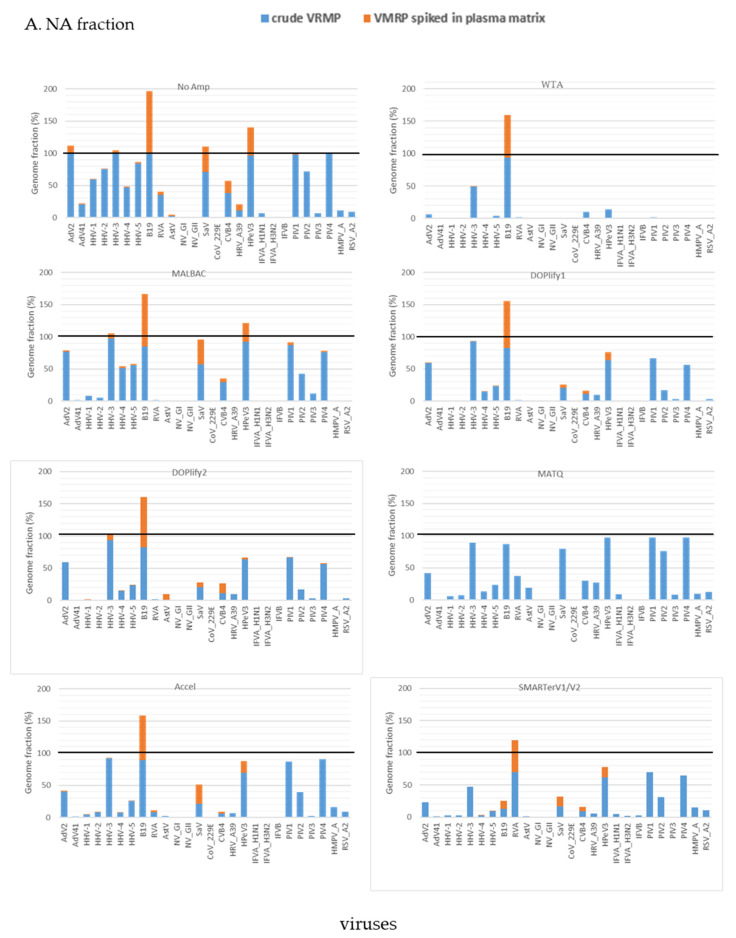

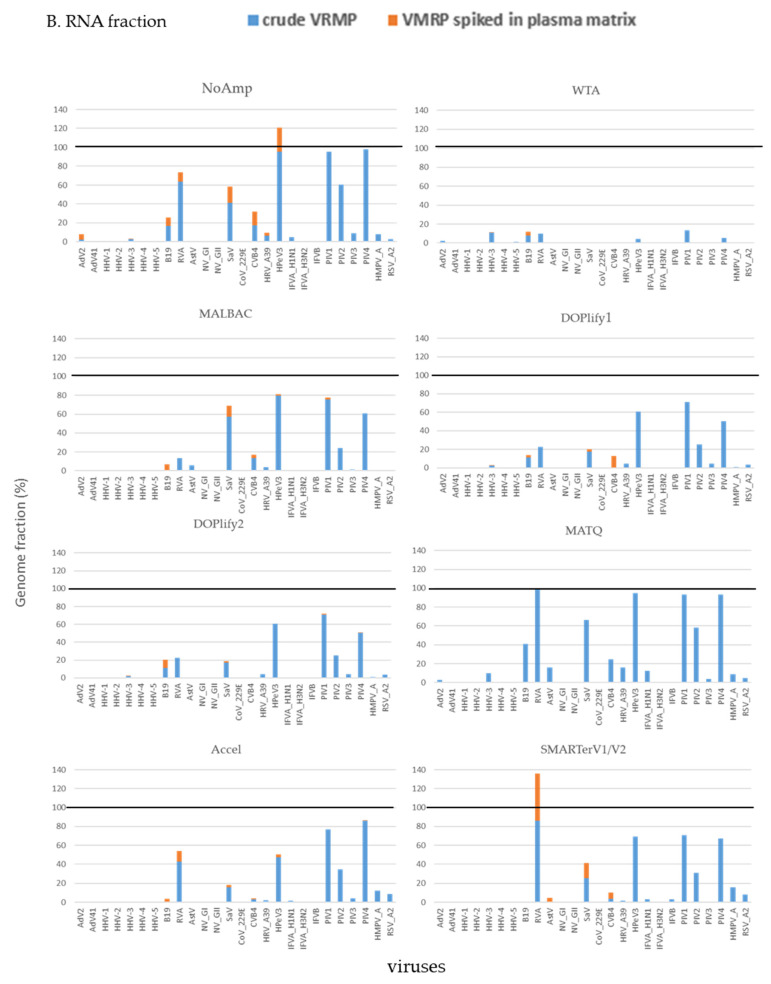
Combined results of virus detection genome fraction of viruses detected in crude VMRP and in VMRP spiked in plasma sample with seven methods and viral detection in VMRP spiked in plasma sample. (**A**) in NA fraction; (**B**) in RNA fraction. Stacked histogram represents the genome fraction (%) in crude VMRP (blue) and in spiked plasma matrix (orange). DOPlify1 and DOPlify2 referred at different number of amplification cycles. Note that MATQ was not assessed in plasma sample.

The genome fraction of viruses detected in VMRP and in VMRP spiked in plasma sample at a volume-ratio 1:10 is compared in Figure 5 for both fractions. In presence of the plasma matrix, increasing the sequencing depth (Table S5) did not allow to detect all viruses present in the crude VMRP, whatever the method (Table S6), suggesting that the increase of sequencing depth was completely consumed by human genetic material.

In the NA fraction, targeted mapping allowed to detect 16 out of 26 viruses of the plasma pool with NoAmp, followed by 9 to 11 viruses with MALBAC, DOPlify and Accel methods, six viruses for SMARTer V2 and only two for WTA (Figure 5A). Virus detection yield was lower in the RNA fraction than in the NA fraction (Figure S6). Due to combined effect of 1:10 dilution and the sequencing of human nucleic acids, some viruses well-covered in PBS became undetectable when they were spiked in the plasma matrix. It was the case for PIV-1, PIV-2 and PIV-4. Only NoAmp, MALBAC and DOP2 detected PIV-1 and PIV-4 in plasma in the NA fraction, but with only very few reads (Table S6).

In the RNA fraction, PIV-1 was only detected by MALBAC, while the other paramyxoviruses were not detected by any other method (Figure 5B). Reads of AstV, not previously detected in the RNA fraction of the raw panel with SMARTerV1, were detected by SMARTerV2 in the plasma matrix. SMARTer lost the ability to detect the *Paramyxoviridae* in presence of the plasma matrix, despite the SMARTer technology depletes libraries containing ribosomal RNA sequences.

Conversely, increasing nucleic acids input and cycles of amplification allowed to detect AstV with the DOPlify method. This was further confirmed by both nucleotide- and protein-based BLAST analyses. Interestingly, AstV was rarely and inefficiently detected by the different methods. In addition, we observed that dsRNA viruses were under-represented, compared to the SMARTer method. We assumed that this could be linked to the denaturation step preceding the reverse transcription. Indeed, dsRNA may be more difficult to denature than ssRNA, resulting in a potentially bias of detection.

### 3.3. Limit of Detection Following Random Amplification Methods in a Complex Biological Matrix

**Figure 6:**
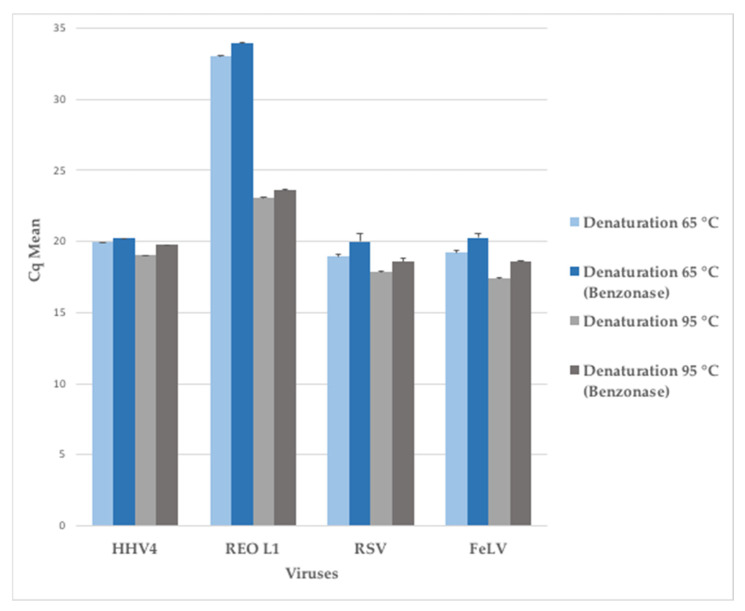
Effect of denaturation temperature of dsRNA virus prior to reverse-transcription on the treated sample with or without benzonase. Each viral stock (HHV-4, REO L1, RSV, FeLV) was diluted to 5 × 10^6^ genome-copies per mL for the NA isolation.

Based on this comparative study, two methods -MATQ and MALBAC- were identified as the most efficient. Indeed, MATQ performed well on the majority of studied criteria (percentage of replicate reads, length of nucleotide contigs, viral genome fraction, identified target ssRNA viruses and WNCS), while MALBAC showed a great potential to amplify viral nucleic acids in the NA fraction. Both methods used MALBAC random primers (Supplementary Materials). Since MATQ was highly performing but time-consuming, we combined a reverse-transcription step derived from the MATQ protocol with a MALBAC amplification step to improve the detection of RNA viruses. In addition, we applied a denaturation modification in the reverse-transcription step to get a better detection of the dsRNA viruses: we tested different temperatures for the denaturation step, from 65 °C to 95 °C (data not shown), and selected the parameters 95 °C for 3 min as the best compromise between the gain in detection of dsRNA viruses and the loss of ssRNA viruses (Figure 6). ssRNA viruses (*Paramyxoviridae*, *Orthoretrovirinae*) were not affected by the increase of temperature while dsRNA viruses (*Reoviridae*) gained more than 3 logs of detection compared to the denaturation at 65 °C. This new protocol was thereafter named MALBAC-V2.

To evaluate the analytical sensitivity of the methods, we compared MALBAC-V2 to the non-modified MALBAC and to the control method NoAmp on WHO reference virus stocks (WRVS) spiked in a plasma matrix at different concentrations (10^2^, 10^3^ and 10^4^ genomes copies/mL). Of note, the agnostic pipeline detected that the HHV-4 stock was contaminated by the squirrel monkey retrovirus (SMRV), expressed by the virus-producer B95-8 cells [32]. SMRV was therefore included in subsequent analyses.

**Figure 7:**
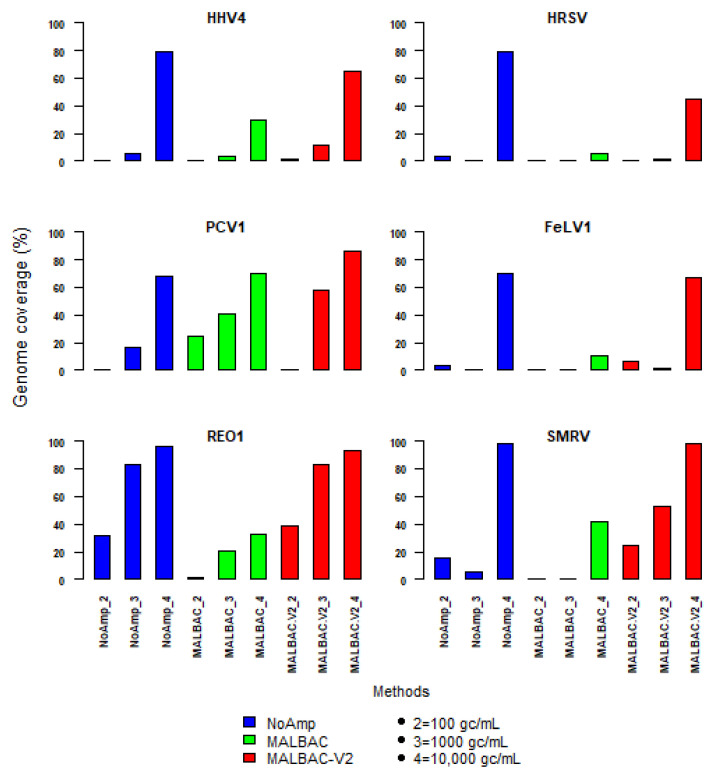
Genome coverage of reference virus stocks (HHV-4, PCV1, REO1, HRSV, FeLV, plus SMRV) spiked in plasma sample pool at 10^2^, 10^3^ or 10^4^ genome-copies per mL (gc/mL) for NoAmp, MALBAC and MALBAC_V2 methods.

The coverage of the viral genomes was assessed by mapping (Figure 7). MALBAC-V2 outperformed MALBAC and was close to the non-pre-amplified method at a spike concentration of 10^4^ gc/mL of plasma. The genome fraction of all viruses was between 50 and 98%. At 10^3^ viral genome copies per mL, the genome coverage was more heterogeneous. PCV1 and REO1 were the best covered viruses, especially with MALBAC-V2. At 10^2^ genomes copies per mL, the genome fraction dropped to less than 10% for all viruses, except for REO1 virus detected with NoAmp and MALBAC-V2.

SMRV, whose concentration is unknown, as a contaminant of HHV4 stock, was also better covered with MALBAC-V2, regardless of the spike level. The coverage depth was more important with MALBAC-V2, but NoAmp showed an even genome coverage profile. This led to get for HHV4 and HRSV a better genome fraction with less viral reads (Table S7). The comparison of genome coverage profiles of the five WRVS viruses spiked in plasma sample at 10^4^ genome-copies per mL is given in Figure S7 for MALBAC-V2 and NoAmp methods. The genome fractions of reads mapping onto each individual segment of REO1, showed differences, not due to the gene length but more likely due to sequence or secondary structures.

## 4. Discussion

Viral spike detection experiments were conducted in order to assess six random pre-amplification methods, with the objective to increase the amount of nucleic acids before library preparation when the starting biological input sample contains a limited amount of viral genetic material. This is often the case when virus genomes enrichment is conducted upstream of nucleic acid extraction. The multiplexed human viral pathogens reagent from NIBSC completed with an ssDNA virus (VMRP) was used as a starting material.

Several studies [31,33,34] reported the use of the NIBSC reagent on different metagenomics methods. Six of the expected 25 viruses were not detected by qPCR [31].

In addition, in order to evaluate the impact of background host nucleic acids on viral detection, the VMRP was diluted into human plasma. After this first step of evaluation, two of the methods were selected, combined, optimized and evaluated using five standard viral stocks endorsed as WHO 1st International Virus Reference Standards for Adventitious Virus Detection in Biological Products by NGS technologies (WRVS). Results were compared to a non-pre-amplification-based method in the NA fraction.

WRVS and VMRP are not highly purified as they originated from cell cultures, egg passages or clinical specimens. The upstream sample processing, including a nuclease pretreatment, inactivation of nuclease before the lysis step and then nucleic acid extraction, was not evaluated in this comparative study, as it was common to all spiking experiments.

The method NoAmp to which we compared the random amplification methods consisted in the reverse-transcription of total RNA into cDNA with random primers, followed by the synthesis of double-stranded DNA from ssRNA/cDNA and ssDNA. Finally, the library was PCR amplified. The method showed the best performance regarding the genome fraction of DNA target viruses and a good one for RNA viruses in the NA fraction, despite usage of a low sequencing depth. The drawback of the method for low nucleic acid input samples is that the concentration of nucleic acids available to construct the libraries is often below the one recommended by manufacturers. As shown in our experiments, it could nevertheless work in such degraded conditions. For example, in this study, the NGS library obtained from VMRP RNA was barely visible on the Bioanalyzer (data not shown). Therefore, it cannot be used in routine with low amounts of starting nucleic acids in conditions that must fulfill quality insurance (diagnostic, testing of pharmaceutical biologics).

The random primer-based MDA procedure used in the WTA method, is based on the use of the highly processive Phi29 polymerase. It produces a huge amount of amplified DNA. However, in our study, it was able to detect the three types of RNA viruses in a DNA-free context (e.g., after conducting a DNase treatment post-extraction of nucleic acids) but was overwhelmingly biased towards dsDNA and ssDNA (*Circoviridae* and *Parvoviridae*) in presence of both DNA and RNA. The systematic over-representation bias of small circular genomes was previously described [24,35,36]. Regarding the number of target viruses detected in this study, we found this method insensitive.

DOPlify and Accel methods gave similar results regarding the horizontal coverage of viral genomes, although based on different principles: Accel-NGS 1S Plus enables the construction of genomic DNA sequencing libraries from either single-stranded and double-stranded DNA and is recommended for highly degraded, damaged or nicked nucleic acids. In our experiments, the ssRNAs were also converted into cDNA with random primers for both fractions followed by the Accel procedure, which also includes the library construction. Accel performed well on ssDNA viruses, but was not very sensitive on dsDNA viruses. The Accel protocol that starts with shearing nucleic acids using the Covaris device does not allow for controlling fragments size before obtaining the final library. DOPlify uses DOP-PCR designed for amplifying total DNA from single cells in a two-step protocol of three hours, with optimized primers and new generation polymerases with high fidelity and proofreading activity compared to the classical DOP-PCR that uses Taq polymerase with a high error rate. The strengths of this method are the amplification efficiency of viral sequences and the ease of use of the kit. The expected sequence accuracy due to the new polymerases was not confirmed in our comparison of the partial sequence of the Phosphoprotein gene of PIV-1 (data not shown). Optimizations would probably be necessary to obtain better detection of viral genomes.

SMARTerV1 and V2 rely on a reverse transcriptase with template-switching activity, and a step enabling removal of ribosomal and mitochondrial cDNA following cDNA synthesis and five cycles of PCR. The method includes the library construction. SMARTer outperformed the other methods in both fractions regarding the detection of dsRNA viruses. Unexpectedly, it performed better on the NA fraction than after a DNase treatment and column purification. The fact that SMARter detected also DNA viruses was very likely due to residual amounts of RNA transcripts in VRMP crude stocks. The rRNA and mtRNA depletion step did not improve the detection of RNA viruses in plasma matrix, compared to other methods without depletion (NoAmp). In addition, the weakness of the protocol lies in the first step, the fragmentation setup. Indeed, the fragmentation parameters are based on the RNA integrity number (RIN), which is obviously not available for the tiny viral fraction among total RNAs). Finally, the protocol does not include a control step before obtaining the library, as for the Accel protocol.

MATQ-seq is a multiple annealing and dC-tailing-based quantitative single-cell RNA-seq, using MALBAC primers for single-cell sequencing of total RNA. The number of detected RNA target viruses in RNA fraction and also their genome fraction were higher with MATQ than with SMARTerV1 for most of them. Despite the good performances, the whole protocol is a time-consuming process. This is the only protocol of this study that is not available as a commercial kit.

MALBAC is based on multiple annealing and looping-based amplification cycles of genomic DNA and cDNA. It utilizes primers containing a 27-nucleotide common sequence and an eight-nucleotide variable sequence to produce fragments of amplified DNA (amplicons) during a quasi-linear pre-amplification step followed by a regular PCR step targeting the conserved sequence. The method generated the highest percentage of viral reads in the NA fraction, the highest sequencing depth and showed a good sequence accuracy, but was not sensitive enough for RNA virus detection. MALBAC and MATQ-seq were selected for further study and combined to ensure the detection of all RNA viruses.

In order to mimic the viral detection within a biological matrix, the VRMP was added to plasma from healthy donors at a ratio 10:1. In these conditions, none of the methods recovered all *Paramyxoviridae* members, even SMARTerV2 that included a removal step of ribosomal and mitochondrial cDNA following cDNA synthesis. NoAmp and MALBAC were the only methods capable of detecting the two viruses that were already the best covered in the absence of plasma (PIV-1 and PIV-4) in the NA fraction. In the RNA fraction the number of RNA viruses detected was smaller than in NA fraction and only MALBAC detected PIV-1. The upstream treatment of plasma samples (low speed centrifugation, and filtration through 0.45 µm membranes) may have played a role in the loss of these viruses, as suggested by Li et al., 2015 [31]. Another explanation of the decrease of single-stranded RNA viruses could be the DNase treatment post extraction to get the DNA-free RNA fraction, which could slightly degrade RNA, or the purification step by column filtration.

DsRNA viruses may be relatively more difficult to denature and biased during reverse-transcription. With a higher temperature for the denaturation step before reverse-transcription, we improved the detection of REO1 with both NoAmp and MALBAC-V2 methods. The upstream treatment and nucleic acid isolation were identical for both methods. Therefore, the improved detection of the dsRNA virus is due to the higher temperature used in the denaturation step before reverse-transcription. The use of the denaturing reagent DMSO prior to cDNA synthesis is also known to increase the dsRNA virus reads [37].

The MALBAC method was first described in 2012 by Zong et al. [38] for Genome-Wide Detection of Single-Nucleotide and Copy-Number Variations of a Single Human Cell and adapted to Single Cell Transcriptome Amplification by Chapman et al., in 2015 [39] to detect copy-number variations and point mutations in the mouse genome. In 2017, Sheng et al. [26] described MATQ-seq for single-cell sequencing of total RNA. In the present study, the MATQ and MALBAC methods were combined and applied to the detection of a wide range of viral genomes in plasma samples at low viral loads.

As performed in MATQ method, reverse-transcription was carried out at low temperature with ten cycles of annealing random MALBAC primers (Supplementary Materials), without oligo (dT) primers. The following step of amplification was carried out using the Yikon’s MALBAC kit. In VMRP experiments, the number of quasi-linear pre-amplification cycles was increased from eight (recommended in the standard protocol) to twelve to get DNA yield above the negative control (water). In VMRP experiments, the number of quasi-linear pre-amplification cycles were restored to standard protocol to reduce the bias associated with non-linear amplification. A fine tuning of the number of quasi-linear pre-amplification cycles could be considered in order to increase the sensitivity of detection of viruses. However, a limitation of MALBAC is the DNA polymerases used that are more error prone than the phi 29 polymerase used in MDA. It can introduce sequencing errors in the first cycle of MALBAC which are subsequently propagated. Indeed, MALBAC uses two relatively error-prone DNA polymerases, the large fragment of *Bacillus stearothermophilus* (Bst) DNA polymerase for isothermal strand displacement and Taq DNA polymerase for PCR [40]. Conversely, the NoAmp method reached its limits using maximal input nucleic acids (11 µL versus 5 µL for MALBAC) and maximal PCR cycles of the libraries. Depending on the viral genome type, the LOD for both methods was either 100 or 1000 genome-copies per mL of virus in such a plasma matrix.

In conclusion, amplification of genomic nucleic acids is a necessary step for the available sequencing technologies and MALBAC-V2 represents a useful method for analysis such biological fluids for low input samples, for example when relative enrichment for viral nucleic acids is conducted before extraction.

## Figures and Tables

**Table 1:** Summary of the methods of amplification and library construction.

Abbreviation	Name	Principle	Library	Comment	Minimum Input Material Recommended	Reference
NoAmp	Control method	Reverse-transcription then double-stranded nucleic acid synthesis	NEBNext® Ultra™ II DNA Library Prep Kit	method with no pre-amplification	500 pg	
WTA	REPLI-g WTA Single Cell Kit (Qiagen)	Multiple displacement amplification (MDA) using phi29 polymerase	NEBNext^®^ Ultra™ II DNA Library Prep Kit		Single Cell	
MALBAC	MALBAC Single Cell WGA Kit (Yikon Genomics)	Multiple annealing and looping-based amplification cycles	NEBNext^®^ Ultra™ II DNA Library Prep Kit	For DNA and cDNA	Single Cell	
DOPlify	DOPlify WGA from RHS (Reproductive Health Science, Thebarton, Australia)	(Degenerate Oligonucleotide Primed) DOP-PCR	NEBNext^®^ Ultra™ II DNA Library Prep Kit	new polymerases or primers compared to classical DOP-PCR	>10 pg	[25]
MATQ	MATQ-Seq	Multiple annealing and dC-tailing-based quantitative single-cell RNA-seq	NEBNext^®^ Ultra™ II DNA Library Prep Kit	For total RNA	Single Cell	[26,27]
Accel	Accel-NGS 1S Plus DNA Library Kit (Swift Biosciences)	For dsDNA and ssDNA.Custom adaptase to ligate adapters to DNA template before PCR.	Included	does not require intact double-stranded DNA	10 pg	
SMARTer V1/V2	SMARTer^®^ Stranded Total RNA-Seq Kit–Pico Input Mammalian. (Takara Bio USA)	Switching Mechanism at the 5′ end of RNA Template	Included	rRNA depletion method after cDNA synthesis	250 pg to 10 ng of total mammalian RNA.	

**Table 2:** DNA yields after pre-amplification and detailed sequencing data from crude VMRP. N/A Not applicable. * 12 cycles of quasi-linear pre-amplification and 25 cycles of amplification.

**Fraction NA**	**Input NA (µL)**	**Qubit DNA (ng) after Pre-Amplification**	**Total Reads PE**	**Percent Duplicate Reads**	**Mapped Reads (26 Viruses)**	**Percent Mapped Reads (26 Viruses)/Total Reads**	**Number of Detected Target Viruses (x/26)**
**NoAmp**	11	<0.5	2,677,664	2.62	36,124	1.35	24
**WTA**	5	4800	1,795,890	11.67	48,844	2.71	13
MALBAC *	5	1180	2,955,706	0.04	612,986	20.70	17
**DOPlify**	4	120	2,194,180	13.5	197,709	9.01	18
**MATQ**	5	323	2,562,750	1.68	15,424	0.60	22
**Accel**	8	N/A	1,781,662	27.13	13,171	0.73	20
**SMARTer**	8	N/A	3,364,988	33.47	12,021	0.35	23
**Fraction RNA**	**Input RNA (µL)**	**Qubit DNA (ng) after Pre-Amplification**	**Total Reads PE**	**Percent Duplicate Reads**	**Mapped Reads (26 Viruses)**	**Percent Mapped Reads (26 Viruses)/Total Reads**	**Number of Detected RNA Target Viruses (x/18)**
**NoAmp**	11	<0.5	991,000	6.78	2654	0.267	11
**WTA**	5	2300	946,624	0	230	0.024	9
**MALBAC**	5	686	3,888,810	3.64	14,331	0.368	10
**DOPlify**	4	50	1,911,114	14.1	36,609	1.915	10
**MATQ**	5	460	2,763,212	1.84	34,149	1.235	15
**Accel**	8	N/A	1,610,944	36.29	2,513	0.155	9
**SMARTer**	8	N/A	3,623,634	53.85	19,672	0.005	11

## Data Availability

The raw data presented in this study and data analyzed with our bioinformatics pipeline are available on request from the corresponding author.

## Supplementary Materials

The following are available online at https://www.mdpi.com/1999-4915/13/2/253/s1.
