# Supplementary material for: Deep Impact of Random Amplification and Library Construction Methods on Viral Metagenomics Results
Source: Viruses. 2021 Feb 7;13(2):253. doi: 10.3390/v13020253 (PMC7915491; doi:10.3390/v13020253)

Supplementary Materials

Tables of contents:

1) Detailed Methods

1. Reverse-transcription
2. NoAmp (non-pre-amplification method)
3. WTA (QuantiTect whole transcriptome kit (Qiagen))
4. DOPlify™ WGA (Reproductive Health Science, Thebarton, Australia)
5. Accel-NGS® 1S Plus DNA Library Kit (Swift Biosciences)
6. The stranded SMARTer technology (Takara Bio/Clontech)
7. The MATQ-seq: multiple annealing and dC-tailing-based quantitative single-cell RNA-seq
8. MALBAC Single Cell WGA kit (Yikon Genomics)
9. MALBAC-V2

2) Supplementary results

Accuracy of the amplification methods

3) Supplementary Tables

Table S1. Virus composition of VMRP (Viral Multiplex Reference NIBSC)
Table S2A. Virus composition of WHO Reference Virus Stocks (WRVS)
Table S2B. Quantification of WHO Reference Virus Stocks (WRVS)
Table S3. Reference viral genomes used for read mapping for WHO Reference Virus Stocks (WRVS)
Table S4: Genome fraction, weighted contigs and singletons and Ct values for each virus of VMRP in both fractions. DNA and RNA viruses are ordered by Ct values.
Table S5: DNA yields after pre-amplification of the fractions NA and RNA and total reads after quality preprocessing for VRMP diluted in plasma (1:10).
Table S6: Genome consensus length of the detected viruses in VRMP diluted in plasma (1:10) for both fractions.
Table S7: Targeted and agnostic analyses of WRVS.

4) Supplementary Figures

Figure S1: Comparison of the distribution of WNCS (log10) detected in the VMRP panel for both NA and RNA fractions for seven methods. Boxes are color-coded according to the methods for both fractions.
Figure S2. Size distribution of contigs in nucleotides generated from *de novo* assembly for the seven methods.
Figure S3. Comparison of cumulative percentage of genome fractions of viruses detected in VMRP with seven methods. (**A**) DNA viruses in RNA and NA fractions. (**B**) RNA viruses in RNA and NA fractions.
Figure S4. Comparison of whole genome coverage profiles of Human parainfluenza virus 1/respirovirus 1 detected in VMRP with six methods in NA and RNA fractions.
Figure S5. Mapping of reads R1 on reference sequence Human parainfluenza virus 1 NC_003461 from Smarter V1 (A) and NoAmp (B) of the NA fractions.
Figure S6. Comparison of genome fraction of VMRP spiked in plasma (ratio 1:10) detected in NA and in RNA fractions, by 100% cumulative bar chart.

Figure S7. Comparison of whole genome coverage profiles of five viruses WRVS spiked in plasma sample at 10^4 genome-copies per mL in NA fraction. For each virus, top profile: MALBAC-V2, bottom profile: NoAmp. Human gammaherpesvirus 4 (HHV-4), porcine circovirus type 1 (PCV1), human orthoreovirus type 1 (REO1), human respiratory syncytial virus strain A2 (HRSV), feline leukemia virus (FeLV), and Squirrel monkey retrovirus (SMRV).

1) Detailed Methods

1. Reverse-transcription

The reverse transcription (RT) using SuperScript® IV First-Strand cDNA Synthesis (Invitrogen) consisted in a mix with 11 µL of RNA (or total nucleic acids), 1 µL of random hexamers (50 µM), and 1 µL of dNTP (10 mM), incubated at 65°C for 5 min and cooled on ice for 2 min, according to the manufacturer's instructions. Seven µL of the RT reaction mix (1X SSIV Buffer, 5mM DTT, 2.0 U Ribonuclease Inhibitor, 10 U SuperScript™ IV Reverse Transcriptase) were added to the 13 µL of annealed RNA and incubated at 23°C for 10 min, at 50°C for 10 min, then inactivated at 80°C for 10 min. To remove RNA, 1 µL *E. coli* RNase H was added, and incubated at 37°C for 20 minutes. Single-stranded cDNA obtained with this protocol was the starting material for NoAmp, WTA, MALBAC, DOP, and Accel protocols for the RNA fraction and the total nucleic acids were also subjected to this protocol for the NA fraction.

For the experiments with WRVS, the denaturation temperature was increased to 95°C for 3 min before cooling on ice for 2 min and used with NoAmp, MALBAC and MALBAC-V2 protocols.

2. NoAmp

Following the reverse transcription step described in section 1, the second cDNA strand was synthetized using a mix containing 1× NEBuffer™ 2, 10 U DNA polymerase I Klenow fragment, 0.33 mM dNTPs , 20 µL of the denatured ss cDNA at 94°C for 2 min, and completed at 30 µL with DNase/RNase-free sterile water. Incubation was done at 37 °C for 1 h. Double-stranded cDNAs were purified using Agencourt AMPure beads (Beckman Coulter).

3. WTA (QuantiTect whole transcriptome kit (Qiagen))

Briefly, the protocol of a QuantiTect whole transcriptome kit (Qiagen) was followed, except that the cDNA synthesis step was performed with random hexamer primers and SuperScript® IV reverse transcriptase (Invitrogen) as described in section 1. The ligation step was performed on 10 µL of the RT reaction followed by the amplification step (6h) using an amplification mix (REPLI-g Midi DNA Polymerase and REPLI-g Midi Reaction Buffer).The resulting dsDNA was purified with the Agencourt AMPure beads (Beckman-Coulter), eluted in a final volume of 50 µL, and quantified with the Qubit™ DNA HS Assay (Life Technologies, Thermo Fisher Scientific Inc.).

4. DOPlify™ WGA (Reproductive Health Science, Thebarton, Australia)

DOPlify™ WGA method used DOP-PCR designed for amplifying total DNA from single cells to in a two-step protocol of three hours. The kit is designed to amplify picogram quantities of DNA. In our experiments, the template for the DOPlify kit was the cDNA synthesis product (1st strand) performed as described in section 1 in a 4 µL volume as input. For the initial amplification at a low annealing temperature, 8 cycles were running. During the second stage of PCR amplification at a higher annealing temperature, 21 cycles were applied. For the viruses spiked into plasma pool, we doubled the volume input and the reaction volumes and ran two assays, either 8/21 cycles or 12/25 cycles for the successive amplifications. The resulting dsDNA was purified with the Agencourt AMPure beads (Beckman-Coulter), eluted and quantified with the Qubit™ DNA HS Assay (Life Technologies, Thermo Fisher Scientific Inc.).

5. Accel-NGS® 1S Plus DNA Library Kit (Swift Biosciences)

DNA fragmentation is the first step to get a peak length of 300 bp by Covaris M220 Focused-ultrasonicator using microTUBE-15 (Peak Incident power (W)=18, Duty Factor=20%, Cycles per Burst =50, Treatment time (sec)=60). Then the Adaptase step was performed on 15 µL of fragmented DNA that simultaneously performs end repair, tailing of 3′ ends, and ligation to the first truncated adapter to 3′ ends in a proprietary reaction. An Extension step is used to facilitate ligation of the second truncated adapter. The synthesized strand does not get sequenced. A double bead-based SPRI clean-up is performed on the extension reaction. The Ligation reaction adds the second truncated adapter to the 5′ ends and the product is cleaned-up. Dual index adapters are added and pre-assembled to the libraries for an amplification with 17 cycles of PCR. The PCR amplified libraries were cleaned-up using 0.9X AmpureXP Beads and concentrated.

6. The stranded SMARTer technology (Takara Bio/Clontech)

The stranded SMARTer technology is based on tagged random hexamer primers and a SMARTScribe Reverse Transcriptase (RT) with terminal transferase activity. When it finishes the first strand cDNA synthesis, the RT adds a few non-templated nucleotides to the 3′ end of the cDNA (GGG). The SMART adapter is complementary to these nucleotides and adds the 5′ tag. The first strand cDNA is used as a matrix to perform the PCR amplification using indexed primers.
A depletion step targeting mammalian rRNA (16S and 28S) and mitochondrial rRNA (m12S and m16S) cut the library fragments using the Zapr enzyme and the remaining library is amplified.
The recommanded quantity of RNA is 0.25–10 ng in a 8 µL volume as input for library preparation using a SMARTer® Stranded Total RNA-Seq Kit v2 - Pico Input Mammalian (Takara Bio/Clontech). Height µL were used as a template with a fragmentation step and 16 cycles or 14 cycles of final RNA-seq library amplification respectively for non-spiked and spiked viruses. Advantages of the version 2 of the SMARTer Stranded Total RNA-Seq Kit version 2 over version 1 announced by the manufacturer is the more efficient removal of rRNA sequences, a higher cDNA yield, improved sequencing performance and Read 2 corresponds to the sense strand.

7. The MATQ-seq: multiple annealing and dC-tailing-based quantitative single-cell RNA-seq

The full protocol is available in (Sheng and Zong, 2019). The MATQ-seq (multiple annealing and dC-tailing-based quantitative single-cell RNA-seq) assay utilizes both oligo dT but also MALBAC random primers to perform first-strand synthesis. For our comparison of methods, based on reverse transcription using random primers, we missed oligo dT. Superscript IV replaced Superscript III reverse transcriptase. To melt secondary structure, samples are incubated at 72°C for 3 min, then 10 thermal cycles of 8°C for 12 s, 15°C for 45 s, 20°C for 45 s, 30°C for 30 s, 42°C for 2 min, 50°C for 3 min, followed by 15 min at 50°C are performed to allow primers to anneal to transcripts. Primers are digested after first-strand synthesis and cDNA is released as single-stranded DNA after a RNA digestion step. The single-stranded cDNA are the polyC tailed on their 3 'end using terminal transferase. MALBAC 6N3G primers bind to the PolyC tail of the single-stranded DNA during the second-strand synthesis using the Deep Vent exo-polymerase (NEB). DNA is then amplified by a 24 cycles of PCR with the GAT27PCR primer. The amplified product was then fragmented to get a peak length of 300 bp by Covaris M220 Focused-ultrasonicator using microTUBE-15 (Peak Incident power (W)=18, Duty Factor=20%, Cycles per Burst =50, Treatment time (sec)=60), then subjected to library preparation using the NEBNext Ultra II DNA Library Prep kit (NEB). The PCR amplified libraries (3 cycles) were cleaned-up using 0.9X AmpureXP Beads.

8. MALBAC Single Cell WGA kit (Yikon Genomics)

MALBAC is based on multiple annealing and looping-based amplification cycles of gDNA and cDNA. The MALBAC Single Cell WGA kit (Yikon Genomics) can be used from 0.5pg of gDNA. It utilizes primers containing a 27 nucleotide common sequence and an height nucleotide variable sequence to produce fragments of amplified DNA (amplicons) during a quasi-linear pre-amplification step followed by a regular PCR step which loop back on themselves to prevent additional copying and cross-hybridization. The common nucleotide sequence is GTG AGT GAT GGT TGA GGT AGT GTG GAG. The template was the cDNA synthesis product (1st strand) performed as described in section 1 in a 5 µL volume as input. In absence of plasma matrix, the quasi-linear pre-amplification step was set on 12 cycles and the regular PCR on 21 cycles and in presence of plasma matrix, we reduced the number of cycles respectively to 8 and 17 cycles.

9. MALBAC-V2

The reverse transcription consisted in a mix with two MALBAC primers (**GAT27 5N3G** and **GAT27 5N3T**) at 5µM each, 0.5nM dNTP, 0.8U RNase inhibitor, 2 mM DTT and 5 µL RNA template. Samples are incubated at 95°C for 3 min and cooled on ice for 2 min. The RT reaction mix (1X SSIV Buffer, 5mM DTT, 1.2 U RNase inhibitor, 10 U SuperScript™ IV Reverse Transcriptase, Invitrogen) as added to the 7 µL of annealed RNA for a total volume of 10 µL. Then 10 thermal cycles of 8°C for 12 s, 15°C for 45 s, 20°C for 45 s, 30°C for 30 s, 42°C for 2 min, 50°C for 3 min, followed by 15 min at 50°C were performed, as in MATQ-seq protocol.

The MALBAC Single Cell WGA kit (Yikon Genomics) is used for the quasi-linear pre-amplification step and the regular amplification step. Freshly-prepared Pre-Amplification Reaction Mix (30µL) is added to 5µL of the reverse transcription product and denatured à 94°c for 3 min. The quasi-linear pre-amplification step consists in 8 to 12 thermal cycles of 20°C for 40 s, 30°C for 40 s, 40°C for 30 s, 50°C for 30 s, 60°C for 30 s, 70°C for 4 min, 95°C for 20 s, 58°C for 10 s and a cooling at 4°C. The freshly-prepared Amplification Reaction Mix (30 µL) was mixed with the 35 µL of the pre-amplified products. The thermocycler conditions are 94°C for 30 s, 17 cycles of 94°C for 20 s, 58°C for 30 s, 72°C for 3 min. The PCR product is then purified using SPRIselect Beads (Beckman Coulter), eluted in a final volume of 20 µL low TE (10 mM Tris-HCl (pH 8,0), 0,1 mM EDTA) and quantified with the Qubit™ DNA HS Assay (Life Technologies, Thermo Fisher Scientific Inc.).

**GAT27 5N3G:**	**GTG AGT GAT GGT TGA GGA TGT GTG GAG NNNNN GGG**
**GAT27 5N3T:**	**GTG AGT GAT GGT TGA GGA TGT GTG GAG NNNNN TTT**
**GAT27 PCR:**	**GTG AGT GAT GGT TGA GGA TGT GTG GAG**

2) Supplementary results

Accuracy of the amplification methods

The assessment of the accuracy of the methods was not the objective of our study, as we were focused on the limit of detection of viruses and did not get a high depth coverage for this purpose. Nevertheless, we indirectly estimated the accuracy of the methods using the general alignment error rate on the human genome from the plasma matrix, computed as a ratio of total collected edit distance to the number of mapped bases with Qualimap 2.2.1 on the BAM alignment (BWA MEM version 0.7.4) against the human reference genome assembly hg38 using VMRP spiked in plasma matrix. In both fractions, the range of this general error rate was between 0.36% and 0.95% for NoAmp, MALBAC, DOP and WTA, with the best result for WTA and reached 1.44%-1.94% for SMARTerV2 and 3.39%-2.08% for Accel. However this result has not been confirmed in the raw VMRP, as the nucleotide sequence alignment (MAFFTv7) of the Phosphoprotein gene of the parainfluenzavirus 1/respirovirus covered by six methods (WTA not used) (594 bp) showed identical variants compared to the reference sequence NC_003461 in both fractions for all methods, except DOP that shared an additional variant in both fractions and another in the RNA fraction (data not shown).

3) Supplementary Tables

Table S1. Virus composition of VMRP (Viral Multiplex Reference NIBSC)

Baltimore classification	Virus Abbrev	Virus name /ICTV	Family	Envelope	Genome size (kb)	PCR Ct value	Sample origin
Group I: dsDNA	AdV2	Human adenovirus 2 / Human mastadenovirus C	*Adenoviridae*	No	35.9	29.71	293 cell culture
	AdV41	Human adenovirus 41 / Human mastadenovirus F	*Adenoviridae*	No	34.2	ND	Clinical specimen
	HHV-1	Human herpesvirus 1 /Human alphaherpesvirus 1	*Herpesviridae*	Yes	151.2	30.59	MRC5 cell culture
	HHV-2	Human herpesvirus 2 /Human alphaherpesvirus 2	*Herpesviridae*	Yes	154.7	32.48	MRC5 cell culture
	HHV-3	Human herpesvirus 3 (VZV) /Human alphaherpesvirus 3	*Herpesviridae*	Yes	124.8	29.02	MeWo cell culture
	HHV-4	Human herpesvirus 4 (EBV) /Human gammaherpesvirus 4	*Herpesviridae*	Yes	171.7	31.27	B95-8 cell culture
	HHV-5	Human herpesvirus 5 (CMV) /Human betaherpesvirus 5	*Herpesviridae*	Yes	233.7	28.95	MRC5 cell culture
Group II: ssDNA	B19	Human parvovirus B19 /Human erythrovirus B19	*Parvoviridae*	No	5.6	24	Plasma
Group III: dsRNA	RVA	Rotavirus A	*Reoviridae*	No	18.5	24.49	Clinical specimen
Group IV: ssRNA (+)	AstV	Human astrovirus /Human astrovirus 1	*Astroviridae*	No	6.8	30.53	Clinical specimen
	NV_GI	Norovirus GI	*Caliciviridae*	No	7.6	ND	Clinical specimen
	NV_GII	Norovirus GII	*Caliciviridae*	No	7.5	ND	Clinical specimen
	SaV	Sapovirus	*Caliciviridae*	No	7.5	33.37	Clinical specimen
	CoV_229E	Human coronavirus 229E	*Coronaviridae*	Yes	27.2	ND	MRC5 cell culture
	CVB4	Human Coxsackievirus B4 /Enterovirus B	*Picornaviridae*	No	7.4	30.72	Hep-2 cell culture
	HRV_A39	Rhinovirus A39 /Enterovirus A	*Picornaviridae*	No	7.1	31.16	MRC5 cell culture
	HPeV3	Parechovirus 3 /Parechovirus A	*Picornaviridae*	No	7.2	29.35	LLC-MK2 cell culture
Group V: ssRNA (-)	IFVA_H1N1	Influenza A virus H1N1	*Orthomyxoviridae*	Yes	13.2	32.02	Egg passage
	IFVA_H3N2	Influenza A virus H3N2	*Orthomyxoviridae*	Yes	13.6	ND	Egg passage
	IFVB	Influenza B virus	*Orthomyxoviridae*	Yes	14.2	ND	Egg passage
	PIV1	Human parainfluenza virus 1 /Human respirovirus 1	*Paramyxoviridae*	Yes	15.5	34.43	PRF5 cell culture
	PIV2	Human parainfluenza virus 2 /Human rubulavirus 2	*Paramyxoviridae*	Yes	15.7	33.87	PRF5 cell culture
	PIV3	Human parainfluenza virus 3 /Human respirovirus 3	*Paramyxoviridae*	Yes	15.4	ND	PRF5 cell culture
	PIV4	Human parainfluenza virus 4 /Human rubulavirus 4	*Paramyxoviridae*	Yes	13.3	31.83	PRF5 cell culture
	HMPV_A	Metapneumovirus A /Human metapneumovirus	*Pneumoviridae*	Yes	17.4	31.86	LLC-MK2 cell culture
	RSV_A2	Respiratory syncytial virus A2 /Human orthopneumovirus	*Pneumoviridae*	Yes	15.2	34.33	Hep-2 cell culture

ND: not detected

Table S2A. Virus composition of WHO Reference Virus Stocks (WRVS)

Virus	Family	virus ATCC	strain	Particle size (nm)	Genome size (kb)	Genome topology	Envelope	Physical-chemical resistance
HHV-4	*Herpesviridae*	Epstein-Barr virus (EBV) /Human herpesvirus 4	strain B95-8 (ATCC ID number SC-VR-6004P)	122-180	180	ds-DNA /circular	yes	low to medium
FeLV	*Orthoretrovirinae*	feline leukemia virus	strain Thielen (ATCC ID number SC-VR-6002P)	80-100	8,448	ss-RNA(+) /dimeric	yes	low
HRSV	*Paramyxoviridae*	human respiratory syncytial virus	strain A2 (ATCC ID number SC-VR-6003P)	150-300	15,158	ss-RNA (−) /linear	yes	low to medium
REO1	*Reoviridae*	human orthoreovirus type 1	strain Lang (ATCC ID number SC-VR-6001P)	60-80	23,5	ds-RNA /segmented(10)	no	medium to high
PCV1	*Circoviridae*	porcine circovirus type [illegible]	ATCC ID number SC-VR-6000P	16-18	1,759	ss-DNA /circular	no	high

Table S2B. Quantification of WHO Reference Virus Stocks (WRVS)

Virus	virus ATCC	infectious virus titer TCID50/mL	genome copy number using digital drop PCR (provided by ATCC). (genome copies/mL)	Ratio genome copies per TCID50/mL
HHV-4	Epstein-Barr virus (HHV-4)	1,10E+07	3,70E+08	3,36E+01
FeLV	feline leukemia virus	2,30E+07	5,30E+10	2,30E+03
HRSV	human respiratory syncytial virus	1,10E+06	1,00E+09	9,09E+02
REO1	human orthoreovirus type 1	1,10E+10	2,40E+09	2,18E-01
PCV1	porcine circovirus type 1	1,20E+07	2,70E+11	2,25E+04

Table S3. Reference viral genomes used for read mapping for WHO Reference Virus Stocks (WRVS)

Virus/Gene	Virus genome length (bp)	Genbank accession number
REO1/ L1	3,854	M24734.1
REO1/ L2	3,915	AF378003.1
REO1/ L3	3,901	AF129820.1
REO1/ M1	2,304	AF461682.1
REO1/ M2	2,203	AF490617.1
REO1/ M3	2,241	AF174382.1
REO1/ S1	1,458	M10260.1
REO1/ S2	1,331	L19774.1
REO1/ S3	1,198	M18389.1
REO1/ S4	1,196	X61586.1

RSV	15,158	JF920069
FeLV	8,448	NC_001940.1
EBV	172,281	V01555
PCV1	1,758	NC_001792.2
SMRV	8,785	M23385

Table S4: Genome fraction, weighted contigs and singletons and Ct values for each virus of VMRP in both fractions. DNA and RNA viruses are ordered by Ct values.

	**Viruses**	**genome fraction (%)**							**Weighted contigs + singletons**							
**Fractions**	**DNA viruses**	**NoAmp**	**WTA**	**MALBAC**	**DOPlify**	**MATQ**	**Accel**	**SMARTerV1**	**NoAmp**	**WTA**	**MALBAC**	**DOPlify**	**MATQ**	**Accel**	**SMARTerV1**	**PCR Ct value**
NA	**B19**	98,78	93,78	85,01	82,65	87,10	89,47	13,24	585497	127043	1318518	1E+06	1E+06	48966	1779	**24,00**
RNA	**B19**	16,71	8,02	**0**	11,29	40,78	**0**	**0**	9210	1323	0	1254	13005	0	0	**24,00**
NA	**CMV**	83,74	3,75	55,67	23,58	23,79	25,68	9,63	1E+06	355030	2,2E+07	2E+06	3E+06	71013	35382	**28.95**
RNA	**CMV**	0,47	1,33	**0**	**0**	0,58	**0**	0,02	1416	5087	0	0	3546	0	0	**28.95**
NA	**VZV**	99,98	49,13	97,49	93,08	88,92	92,22	47,63	4E+06	2E+06	2,8E+07	1E+07	1E+07	557412	168597	**29.02**
RNA	**VZV**	2,77	10,70	**0**	2,23	10,06	**0**	**0**	4482	25117	0	3574	30065	0	0	**29.02**
NA	**AdV2**	99,95	6,40	77,17	59,38	42,15	40,45	22,92	386512	128871	7428519	1E+06	1E+06	17730	10926	**29.71**
RNA	**AdV2**	2,47	2,39	**0**	0,45	2,76	**0**	0,30	0	1662	0	312	0	0	0	**29.71**
NA	**HHV1**	59,48	0,11	7,96	0,50	5,81	4,74	2,55	532029	4320	3059492	46670	2E+06	9855	8508	**30.59**
RNA	**HHV1**	**0**	**0**	**0**	**0**	**0**	**0**	**0**	0	609	0	0	0	0	0	**30.59**
NA	**EBV**	46,80	1,05	51,57	14,67	13,14	7,34	2,70	184992	5954	1,5E+07	608215	1E+06	11718	4422	**31.27**
RNA	**EBV**	**0**	0,56	**0**	**0**	0,67	**0**	0,04	0	1014	0	0	1167	0	0	**31.27**
NA	**HHV2**	75,03	0,11	5,17	0,97	7,64	7,89	2,51	552237	2161	1350780	2745	1E+06	8598	4293	**32.48**
RNA	**HHV2**	**0**	0,80	**0**	**0**	0,14	**0**	**0**	0	1698	0	0	693	0	0	**32.48**
NA	**AdV41**	20,69	0	2,01	0	0	1,92	1,32	16371	0	671494	0	12942	345	567	**ND**
RNA	**AdV41**	**0**	**0**	**0**	**0**	**0**	**0**	**0**	0	0	0	0	0	0	0	**ND**
		**genome fraction (%)**							**Weighted contigs + singletons**							
**Fractions**	**RNA viruses**	**No Amp**	**WTA**	**MALBAC**	**DOPlify**	**MATQ**	**Accel**	**SMARTerV1**	**NoAmp**	**WTA**	**MALBAC**	**DOPlify**	**MATQ**	**Accel**	**SMARTerV1**	**PCR Ct value**
NA	**RVA**	34,86	1,61	1,70	1,47	37,11	8,45	70,03	107517	486	612	8058	685016	1419	329512	**24.49**
RNA	**RVA**	63,65	9,83	13,52	22,55	98,97	42,66	85,86	78403	22164	376596	350260	4E+07	20772	937325	**24.49**
NA	**HPeV3**	96,69	14,14	92,56	63,74	97,24	69,49	62,19	166846	9156	1399692	281515	2E+06	5175	15090	**29.35**
RNA	**HPeV3**	95,10	4,35	79,75	60,78	94,86	47,57	69,07	77122	642	747603	284627	914471	3093	4407	**29.35**
NA	**AstV**	2,24	0	0	0	19,51	2,17	1,00	300	0	0	0	48487	72	276	**30.53**
RNA	**AstV**	**0**	**0**	5,61	**0**	16,04	**0**	**0**	0	0	47838	0	2100	0	0	**30.53**
NA	**CVB4**	38,33	10,03	29,35	10,91	30,40	6,21	9,71	8757	2160	207837	68223	78477	429	189	**30.72**
RNA	**CVB4**	17,60	**0**	13,22	**0**	24,71	2,97	3,46	1008	0	3105	0	246168	0	0	**30.72**
NA	**HRV_A39**	11,36	0	0	9,86	27,01	7,16	6,00	546	0	0	13815	1884	570	210	**31.16**
RNA	**HRV_A39**	6,64	**0**	3,99	4,30	16,14	1,96	1,75	690	0	423	37923	112728	0	276	**31.16**
NA	**PIV4**	99,61	1,36	76,53	56,58	97,33	90,48	64,98	323775	288	3002924	685379	3E+06	71181	36525	**31.83**
RNA	**PIV4**	97,76	5,39	61,03	50,23	93,26	85,80	67,39	176686	1326	1279353	419603	5E+06	25086	35325	**31.83**
NA	**HMPV_A**	10,86	0	0	1,05	9,55	16,00	15,07	37100	0	0	116685	33712	4329	4155	**31.86**
RNA	**HMPV_A**	8,02	**0**	**0**	0,97	9,00	12,24	15,88	3024	0	0	24672	200442	2577	10200	**31.86**
NA	**IFVA_H1N1**	6,83	0	0	0	8,70	0	4,78	3669	0	0	0	61832	0	1467	**32.02**
RNA	**IFVA_H1N1**	4,95	**0**	**0**	**0**	12,35	1,63	3,19	180	0	0	0	489958	0	138	**32.02**
NA	**SaV**	70,78	0	56,84	20,69	79,47	21,31	16,92	244002	10660	3738950	535891	3E+06	30672	20622	**33.37**
RNA	**SaV**	41,47	**0**	57,15	17,24	66,35	15,67	25,33	125172	2268	2871744	390263	8E+06	19653	48126	**33.37**
NA	**PIV2**	71,87	0	42,77	16,99	76,38	39,31	31,56	47874	0	1160043	199624	1E+06	9096	10233	**33.87**
RNA	**PIV2**	60,72	0,81	24,13	25,30	58,34	34,86	31,31	31476	246	301161	388379	2E+06	9642	20715	**33.87**
NA	**RSV_A2**	8,64	0	0,00	3,23	12,36	9,26	10,97	3822	0	98406	447	8488	996	1233	**34.33**
RNA	**RSV_A2**	3,00	**0**	**0**	3,52	4,50	8,49	8,06	0	0	0	61563	4128	846	948	**34.33**
NA	**PIV1**	98,23	1,58	87,35	66,72	97,02	87,13	69,65	542099	468	3267668	1E+06	2E+06	72528	69579	**34.43**
RNA	**PIV1**	95,51	13,22	75,60	71,13	93,21	76,69	70,83	258170	3693	2286864	1E+06	8E+06	61248	129702	**34.43**
NA	**PIV3**	6,42	0	12,03	3,00	8,61	2,28	0	8927	0	400217	2079	857181	726	132	**ND**
RNA	**PIV3**	8,81	**0**	1,04	4,32	3,64	4,16	0,47	3540	0	5190	73653	424034	1347	0	**ND**
NA	**CoV_229E**	1,15	0	0	0	0	0	0,15	630	0	0	0	0	0	0	**ND**
RNA	**CoV_229E**	**0**	**0**	**0**	**0**	**0**	**0**	**0**	0	0	0	0	0	0	0	**ND**
NA	**IFVA_H3N2**	0	0	0	0	0,59	0	1,73	0	0	0	0	0	0	50	**ND**
RNA	**IFVA_H3N2**	**0**	**0**	**0**	**0**	**0**	0,51	**0**	0	0	0	0	102	0	0	**ND**
NA	**IFVB**	0,57	0	0	0	0	0	2,74	162	0	0	0	22	0	555	**ND**
RNA	**IFVB**	**0**	**0**	**0**	**0**	**0**	**0**	3,13	0	0	0	0	153	0	1101	**ND**
NA	**NV_GI**	0	0	0	0	0	0	0	0	0	0	0	0	0	0	**ND**
RNA	**NV_GI**	**0**	**0**	**0**	**0**	**0**	**0**	**0**	0	0	0	0	0	0	0	**ND**
NA	**NV_GII**	0	0	0	0	0	0	0	684	0	0	0	0	0	0	**ND**
RNA	**NV_GII**	**0**	**0**	**0**	**0**	**0**	**0**	**0**	0	0	0	0	0	0	0	**ND**

Table S5: DNA yields after pre-amplification of the fractions NA and RNA and total reads after quality preprocessing for VRMP diluted in plasma (1:10).

VMRP+Plasma	Input NA or RNA (µL)	Qubit DNA (ng) after pre-amplification	total reads PE
**Fraction NA**			
**NoAmp**	11	14	47,533,260
**WTA**	5	2500	24,488,418
**MALBAC [1]**	5	79,8	35,553,024
**DOPlify1***	8	204	24,475,408
**DOPlify2***	8	98,4	31,465,452
**Accel**	8	Not applicable	31,688,834
**SMARTer V2**	8	Not applicable	17,243,974
SD			9,712,616
**Fraction RNA**			
**NoAmp**	11	26,6	14,252,900
**WTA**	5	1024	17,604,150
**MALBAC**	5	64,8	22,732,152
**DOPlify1***	8	62,6	34,062,016
**DOPlify2***	8	56	30,467,000
**Accel**	8	Not applicable	27,285,343
**SMARTer V2**	8	Not applicable	21,368,064
SD			7,051,001
MALBAC [1]		8/17 cycles	
DOPlify1*		12/25 cycles	
DOPlify2*		8/21 cycles	

Table S6: Genome consensus length of the detected viruses in VRMP diluted in plasma (1:10) for both fractions.

Virus	Reference length (bp)	Consensus length (bp)													
Method Fractions: NA or RNA		NoAmp NA	NoAmp RNA	WTA NA	WTA RNA	MALBAC NA	MALBAC RNA	DOPlify1 NA	DOPlify1 RNA	DOPlify2 NA	DOPlify2 RNA	SMARTer V2 NA	SMARTer V2 RNA	Accel NA	Accel RNA
AdV2	35937	4137	2054	0	0	791	0	359	0	0	0	0	0	377	0
AdV41	34138	367	0	0	0	0	0	0	0	0	0	0	254	0	0
HHV-1	152261	856	440	0	0	0	0	128	106	225	0	0	0	617	149
HHV-2	154675	379	0	0	0	0	0	0	0	0	0	0	0	106	0
HHV-3	124884	5367	264	268	235	9510	334	600	268	11638	374	0	0	1043	230
HHV-4	172764	2418	108	0	0	5171	0	348	0	93	0	759	0	511	0
HHV-5	235646	5108	639	0	0	5810	0	2124	0	687	0	1443	0	1239	0
B19	5596	5483	506	3685	212	4571	392	4093	148	4338	497	691	0	3871	212
RVA	18038	956	1772	0	0	0	0	0	0	0	0	8977	9062	532	2040
AstV	6771	144	0	0	0	0	0	0	0	631	0	0	310	0	0
NV_GI	7654	0	0	0	0	0	0	0	0	0	0	0	0	0	0
NV_GII	7567	0	0	0	0	0	0	0	0	0	0	0	0	0	0
SaV	7429	2925	1278	0	0	2908	881	366	212	530	106	1107	1194	2231	204
CoV_229E	27317	0	0	0	0	0	0	0	0	0	0	0	0	0	0
CVB4	7397	1374	1068	0	0	462	274	417	929	1165	0	439	495	203	101
HRV_A39	7137	635	192	0	0	0	0	0	0	0	0	0	0	0	0
HPeV3	7349	3210	1874	0	0	2124	106	894	0	184	0	1171	0	1329	203
IFVA_H1N1	13588	0	0	0	0	0	0	0	0	0	0	0	0	0	0
IFVA_H3N2	13627	0	0	0	0	0	0	0	0	0	0	0	0	0	0
IFVB	14452	0	0	0	0	0	0	0	0	0	0	0	0	0	0
PIV1	15600	217	0	0	0	652	304	0	0	106	106	0	0	0	0
PIV2	15646	0	0	0	0	0	0	0	0	0	0	0	0	0	0
PIV3	15409	0	0	0	0	0	0	0	0	0	0	0	0	0	0
PIV4	17304	106	0	0	0	381	0	0	0	211	105	0	0	0	102
HMPV_A	13251	0	0	0	0	0	0	0	0	0	0	0	0	0	0
RSV_A2	15191	0	0	0	0	0	0	0	0	0	0	0	0	0	0

Table S7: Targeted and agnostic analyses of WRVS: Total viral read count, genome fraction; average coverage for the targeted approach and WNCS for the agnostic approach.

Analysis	Targeted approach						Agnostic approach	
Methods	NoAmp 10,000 gc/mL	MALBAC-V2 10,000 gc/mL	NoAmp 10,000 gc/mL	MALBAC-V2 10,000 gc/mL	NoAmp 10,000 gc/mL	MALBAC-V2 10,000 gc/mL	NoAmp 10,000 gc/mL	MALBAC-V2 10,000 gc/mL
**Virus**	**Total read count (R1)**		**Genome fraction (%)**		**Average coverage**		**WNCS**	
**HHV-4**	2062	2571	74.29	65.57	1.63	2.61	260355	9764774
**PCV1**	22	96	63.77	86.06	1.65	9.73	1557	12450
**REO1**	5810	5644	95.98	95.36	35.10	42.59	620357	1944510
**HRSV**	146	208	71.63	47.49	1.38	2.31	10134	213123
**FeLV**	104	147	66.96	70.02	1.67	2.91	10254	359760
**SMRV**	1516	1381	97.73	98.29	ND	ND	195381	864080
Methods	NoAmp 1000 gc/mL	MALBAC-V2 1000 gc/mL	NoAmp 1000 gc/mL	MALBAC-V2 1000 gc/mL	NoAmp 1000 gc/mL	MALBAC-V2 1000 gc/mL	NoAmp 1000 gc/mL	MALBAC-V2 1000 gc/mL
**Virus**	**Total read count (R1)**		**Genome fraction (%)**		**Average coverage**		**WNCS**	
**HHV-4**	154	219	5.94	11.67	0.10	0.17	7731	357273
**PCV1**	1	10	16.95	60.07	0.17	0.84	291	166539
**REO1**	706	2272	89.26	87.87	6.37	16.50	141138	1393055
**HRSV**	1	4	0.23	1.87	0.00	0.02	0	108
**FeLV**	0	13	0	3.60	0	0.10	0	2134
**SMRV**	2	53	4.54	53.30	ND	ND	455	132948
Methods	NoAmp 100 gc/mL	MALBAC-V2 100 gc/mL	NoAmp 100 gc/mL	MALBAC-V2 100 gc/mL	NoAmp 100 gc/mL	MALBAC-V2 100 gc/mL	NoAmp 100 gc/mL	MALBAC-V2 100 gc/mL
**Virus**	**Total read count (R1)**		**Genome fraction (%)**		**Average coverage**		**WNCS**	
**HHV-4**	147	183	0.87	2.30	0.050	0.059	225	2559
**PCV1**	1	0	1.37	0	0.013	0	0	0
**REO1**	49	130	32.44	41.40	0.460	0.998	5100	296666
**HRSV**	4	2	3.75	0.35	0.052	0.004	330	0
**FeLV**	3	17	4.24	8.77	0.042	0.172	129	75357
**SMRV**	10	19	14.96	24.34	ND	ND	2781	136761

Supplementary Figures

Figure S1: Comparison of the distribution of WNCS (log10) detected in the VMRP panel for both NA and RNA fractions for seven methods. Boxes are color-coded according to the methods for both fractions. Boxes represent the interquartile range, bar the median value, and circles the outliers.

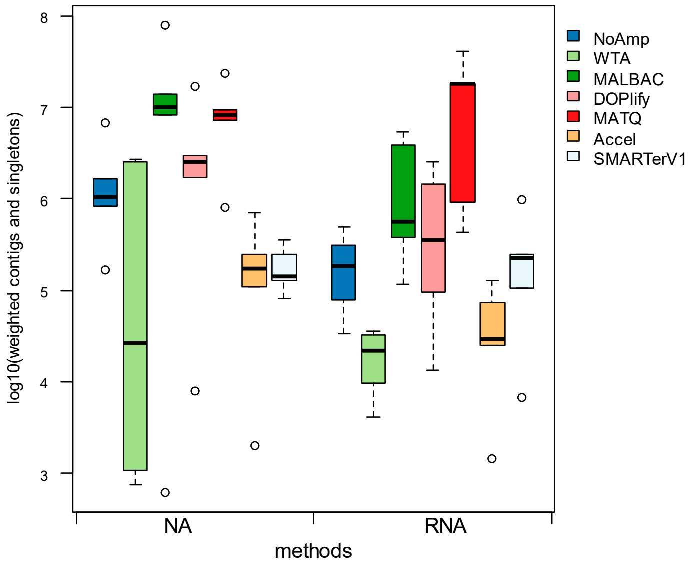

Figure S2. Size distribution of contigs in nucleotides generated from *de novo* assembly for the seven methods. Boxes represent the interquartile range, bar the median value, and dots the outliers.

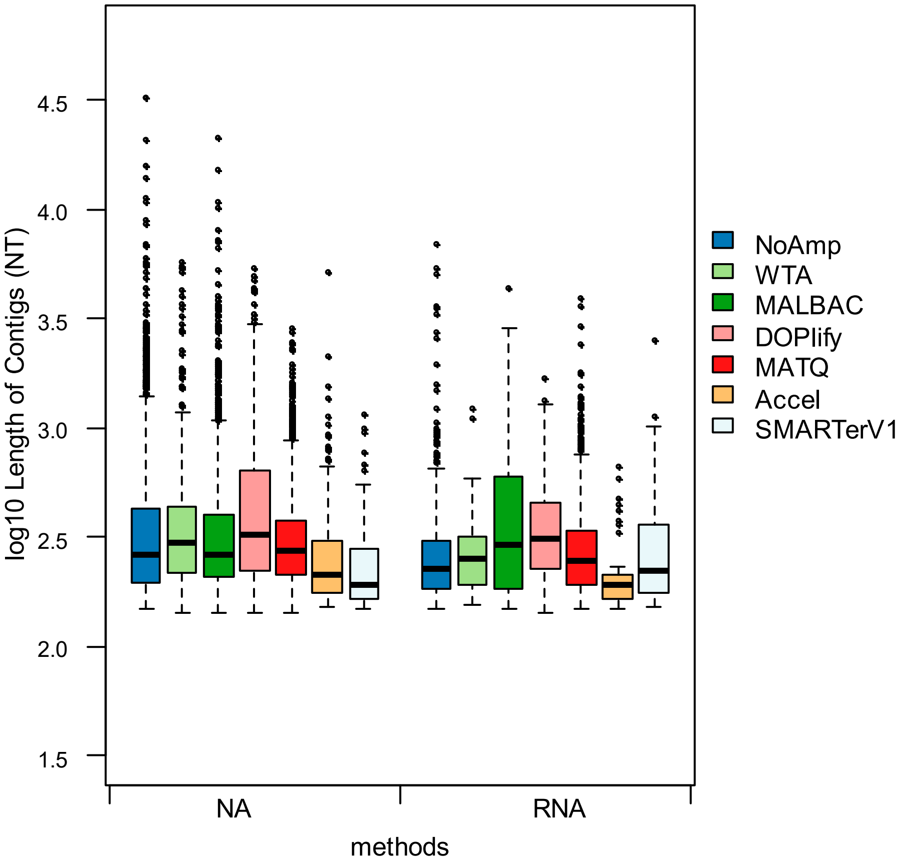

Figure S3. Comparison of cumulative percentage of genome fractions of viruses detected in VMRP with seven methods. (**A**) DNA viruses in RNA and NA fractions. (**B**) RNA viruses in RNA and NA fractions.

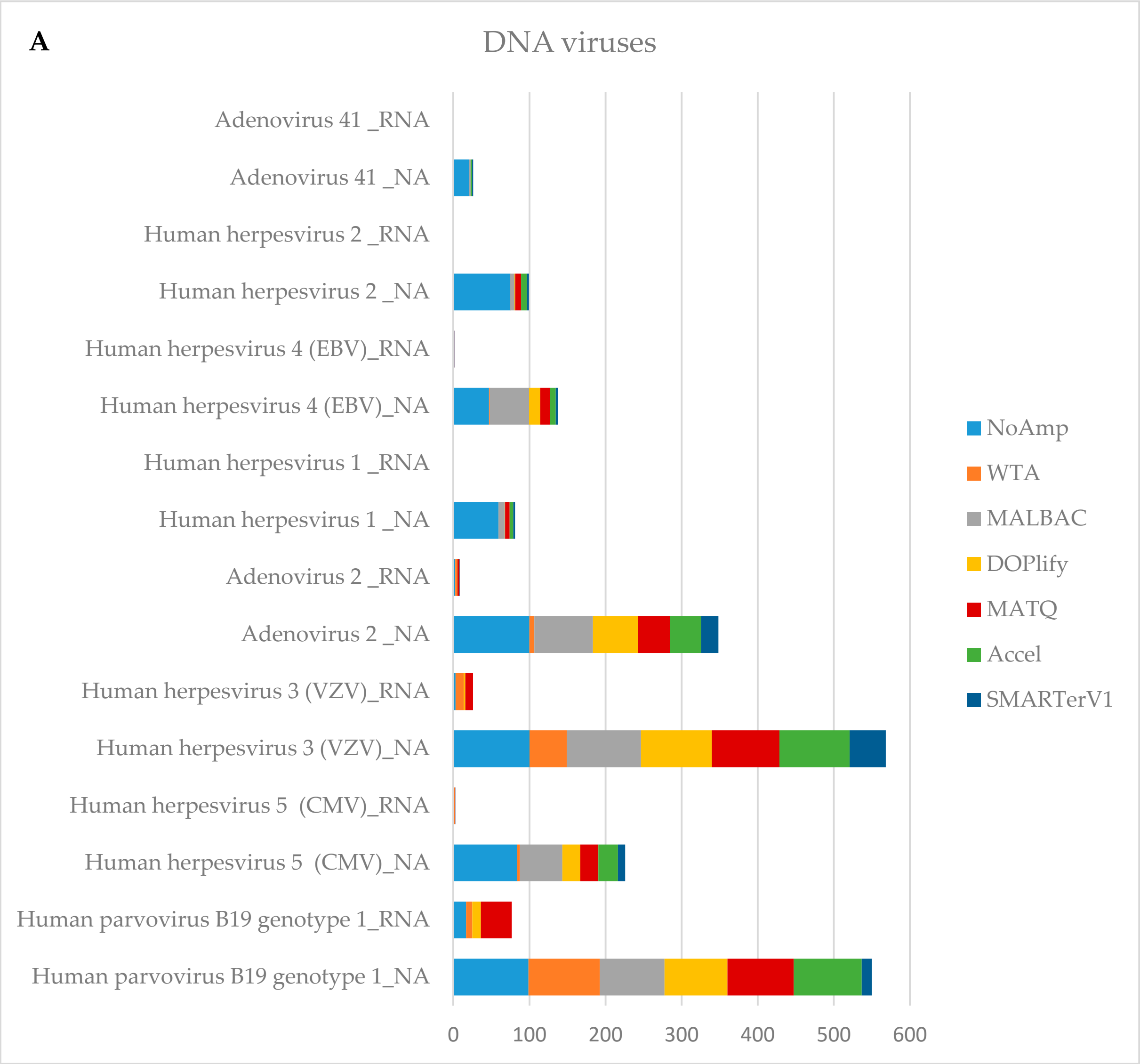

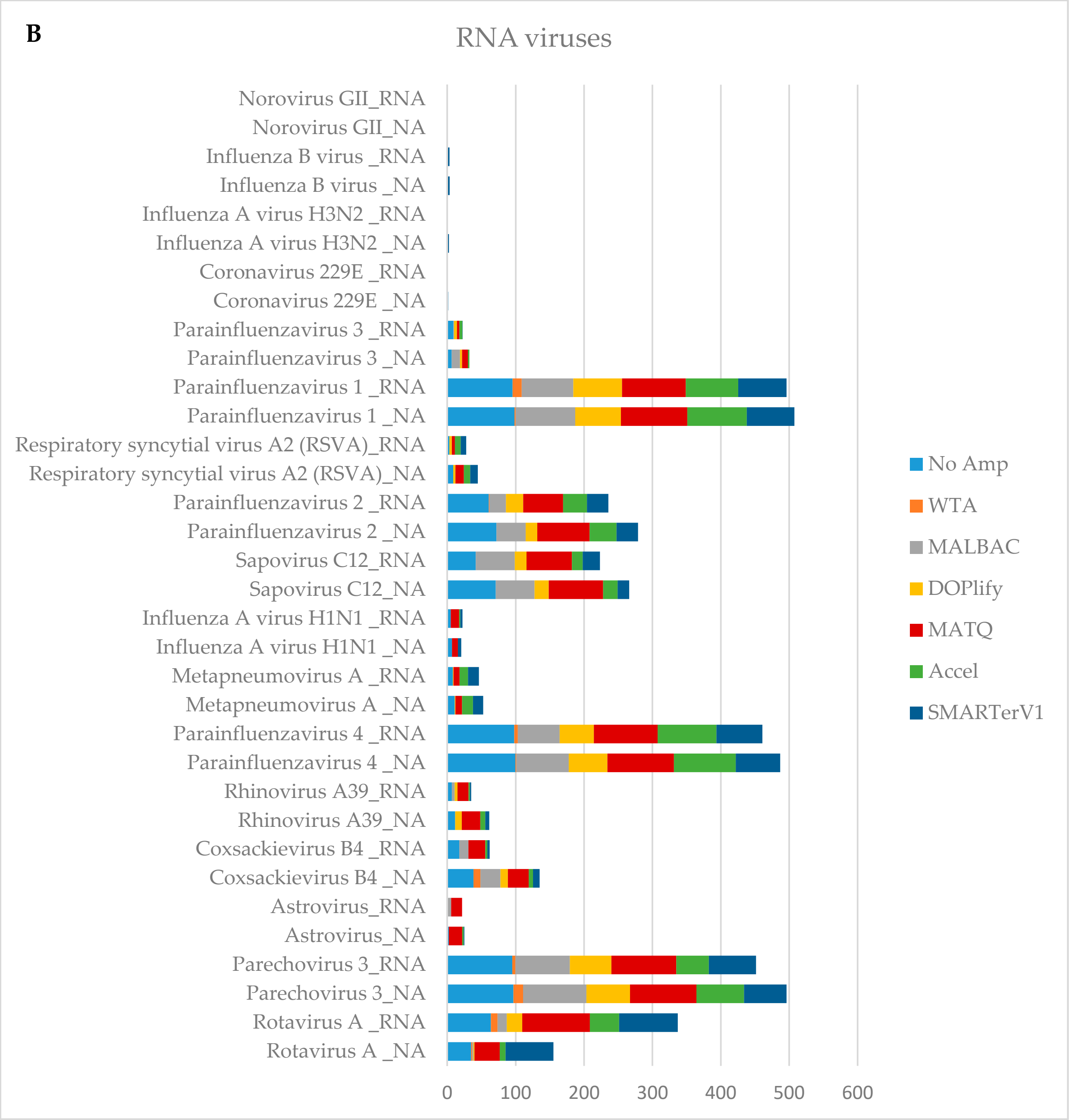
B
RNA viruses
Norovirus GII_RNA
Norovirus GII_NA
Influenza B virus _RNA
Influenza B virus _NA
Influenza A virus H3N2 _RNA
Influenza A virus H3N2 _NA
Coronavirus 229E _RNA
Coronavirus 229E _NA
Parainfluenzavirus 3 _RNA
Parainfluenzavirus 3 _NA
Parainfluenzavirus 1 _RNA
Parainfluenzavirus 1 _NA
Respiratory syncytial virus A2 (RSVA)_RNA
Respiratory syncytial virus A2 (RSVA)_NA
Parainfluenzavirus 2 _RNA
Parainfluenzavirus 2 _NA
Sapovirus C12_RNA
Sapovirus C12_NA
Influenza A virus H1N1 _RNA
Influenza A virus H1N1 _NA
Metapneumovirus A _RNA
Metapneumovirus A _NA
Parainfluenzavirus 4 _RNA
Parainfluenzavirus 4 _NA
Rhinovirus A39_RNA
Rhinovirus A39_NA
Coxsackievirus B4 _RNA
Coxsackievirus B4 _NA
Astrovirus_RNA
Astrovirus_NA
Parechovirus 3_RNA
Parechovirus 3_NA
Rotavirus A _RNA
Rotavirus A _NA
0
100
200
300
400
500
600
No Amp
WTA
MALBAC
DOPlify
MATQ
Accel
SMARTerV1

Figure S4. Comparison of whole genome coverage profiles of Human parainfluenza virus 1/respirovirus 1 detected in VMRP with six methods in NA and RNA fractions.

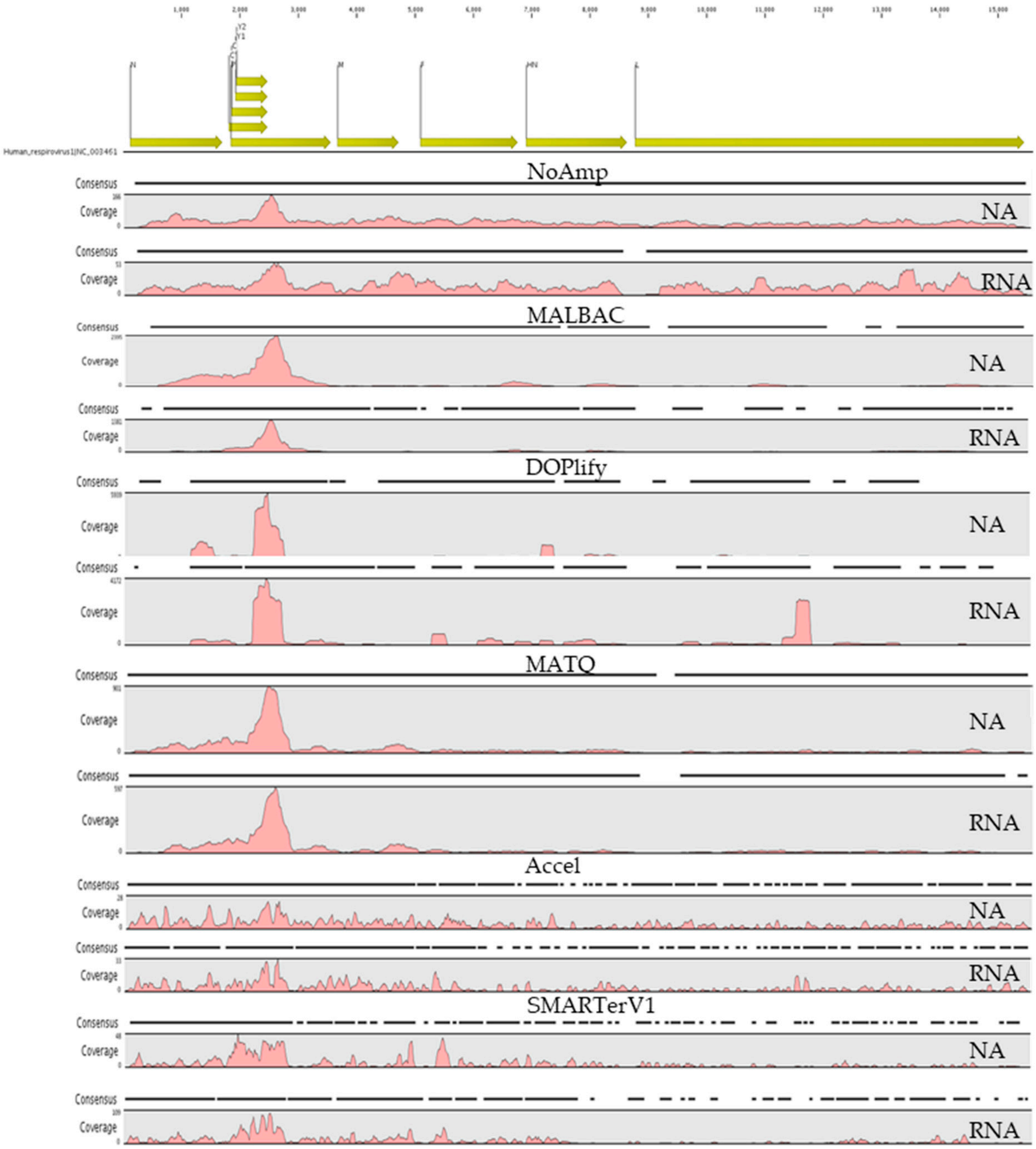

Figure S5. Mapping of reads R1 on reference sequence Human parainfluenza virus 1 NC_003461 from Smarter V1 (A) and NoAmp (B) of the NA fractions.

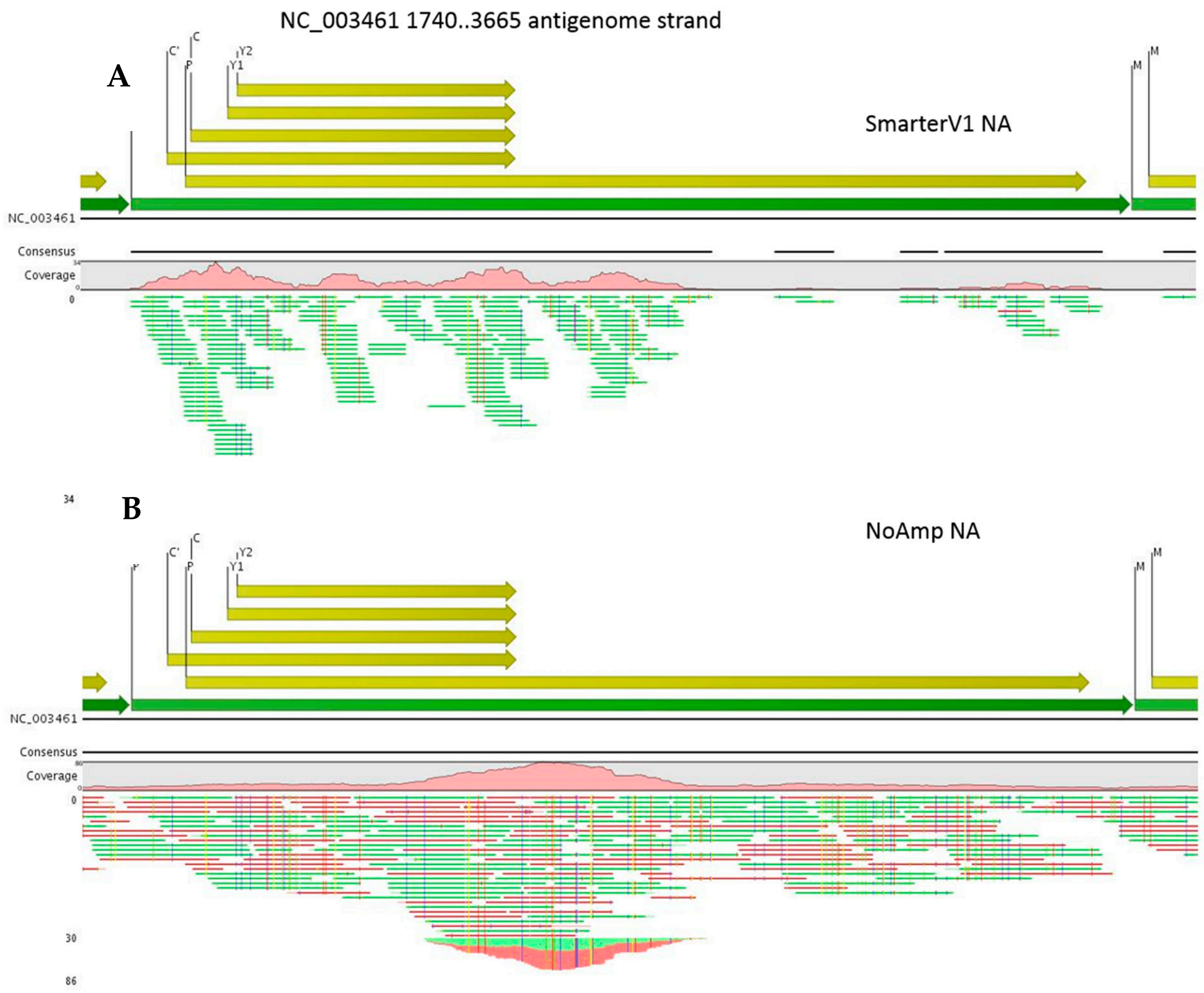

Figure S6. Comparison of genome fraction of VMRP spiked in plasma (ratio 1:10) detected in NA (blue) and in RNA (orange) fraction by 100% stacked bar chart.

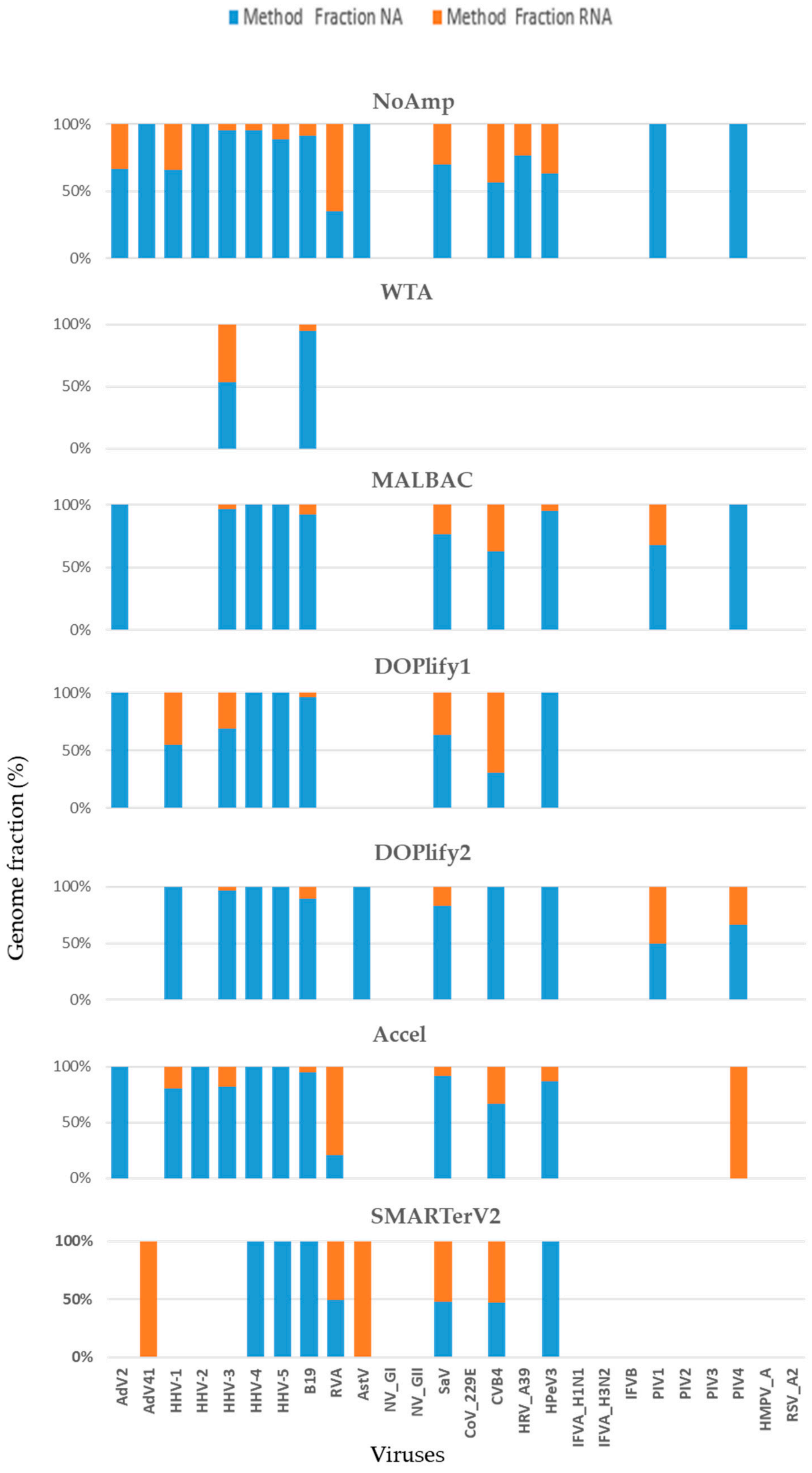

Figure S7. Comparison of whole genome coverage profiles of five viruses WFRVS spiked in plasma sample at 10^4 genome-copies per mL in NA fraction. For each virus, top profile: MALBAC-RNAV2, bottom profile: NoAmp. Human gammaherpesvirus 4 (HHV-4), porcine circovirus type 1 (PCV1), human orthoreovirus type 1 (REO1), human respiratory syncytial virus strain A2 (HRSV), feline leukemia virus (FeLV), and Squirrel monkey retrovirus (SMRV).

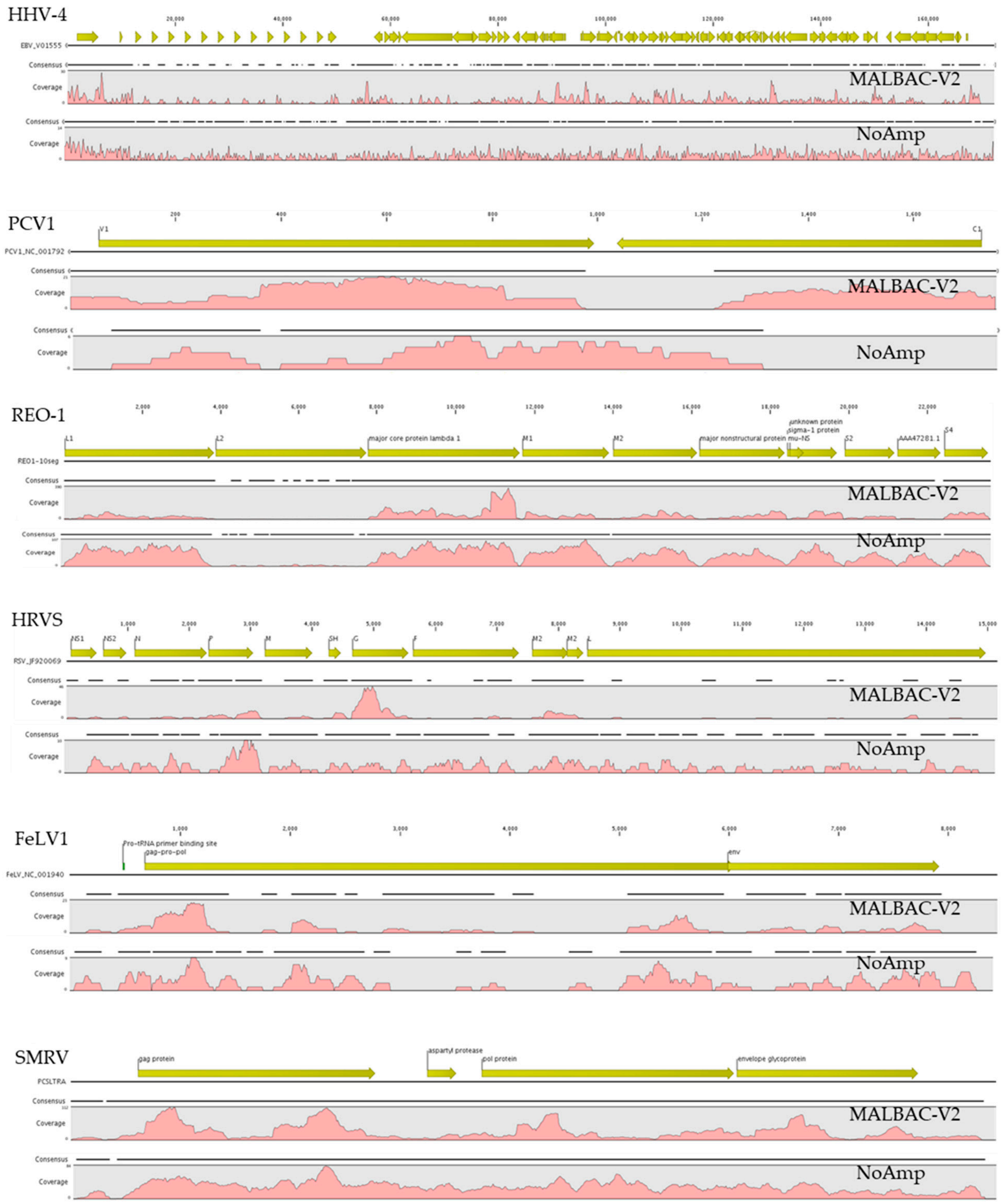

Supplement: Supplementary file 1 [file viruses-13-00253-s001.pdf]
